# Putative adverse outcome pathways for silver nanoparticle toxicity on mammalian male reproductive system: a literature review

**DOI:** 10.1186/s12989-022-00511-9

**Published:** 2023-01-05

**Authors:** Ozge Kose, Paride Mantecca, Anna Costa, Marie Carrière

**Affiliations:** 1 Univ. Grenoble-Alpes, CEA, CNRS, IRIG, SyMMES-CIBEST, 38000 Grenoble, France; 2 Polaris Research Centre, Department of Earth and Environmental Sciences, University of Milano-Bicocca, Piazza della Scienza, 1, 20126 Milan, Italy; 3 CNR-ISTEC, Institute of Science and Technology for Ceramics-National Research Council of Italy, Via Granarolo 64, 48018 Faenza, Italy

**Keywords:** Adverse outcome pathways, Silver nanoparticles, Reproductive toxicity, Molecular initiating event, Key events, Adverse outcome

## Abstract

**Background:**

Adverse outcome pathways (AOPs) are conceptual frameworks that organize knowledge about biological interactions and toxicity mechanisms. They present a sequence of events commencing with initial interaction(s) of a stressor, which defines the perturbation in a biological system (molecular initiating event, MIE), and a dependent series of key events (KEs), ending with an adverse outcome (AO). AOPs have recently become the subject of intense studies in a view to better understand the mechanisms of nanomaterial (NM) toxicity. Silver nanoparticles (Ag NPs) are one of the most explored nanostructures and are extensively used in various application. This, in turn, has increased the potential for interactions of Ag NPs with environments, and toxicity to human health. The aim of this study was to construct a putative AOPs (pAOP) related to reproductive toxicity of Ag NPs, in order to lay the groundwork for a better comprehension of mechanisms affecting both undesired toxicity (against human cell) and expected toxicity (against microorganisms).

**Methods:**

PubMed and Scopus were systematically searched for peer-reviewed studies examining reproductive toxicity potential of Ag NPs. The quality of selected studies was assessed through ToxRTool. Eventually, forty-eight studies published between 2005 and 2022 were selected to identify the mechanisms of Ag NPs impact on reproductive function in human male. The biological endpoints, measurements, and results were extracted from these studies. Where possible, endpoints were assigned to a potential KE and an AO using expert judgment. Then, KEs were classified at each major level of biological organization.

**Results:**

We identified the impairment of intracellular SH-containing biomolecules, which are major cellular antioxidants, as a putative MIE, with subsequent KEs defined as ROS accumulation, mitochondrial damage, DNA damage and lipid peroxidation, apoptosis, reduced production of reproductive hormones and reduced quality of sperm. These successive KEs may result in impaired male fertility (AO).

**Conclusion:**

This research recapitulates and schematically represents complex literature data gathered from different biological levels and propose a pAOP related to the reproductive toxicity induced by AgNPs. The development of AOPs specific to NMs should be encouraged in order to provide new insights to gain a better understanding of NP toxicity.

**Supplementary Information:**

The online version contains supplementary material available at 10.1186/s12989-022-00511-9.

## Background

With respect to the European recommendation on the definition for nanomaterials (NMs), adopted in 2011 (Recommendation 2011/696/EU) and revised in 2022 (2022/C 229/01), NMs are materials with at least one dimension ranging between 1 and 100 nm, except those with a specific surface area by volume < 6 m^2^/cm^3^ [1]. Despite having the same composition as the corresponding bulk material, due to size effects, NPs display distinct characteristics. Among the NMs, Silver nanoparticles (Ag NPs) are one of the most studied ones and they have been extensively used in various fields such as in food, cosmetic, textile, and medical industries [2–4]. Their high preference in such areas is generally attributed to their unique features differing from bulk materials, including optical, electrical, magnetic and antibacterial properties [5, 6]. Given that Ag NPs are incorporated into commercially available products primarily for antibacterial and antifungal purposes, their abundance in everyday products leads to concerns related to their health effects and the possible consequences of their dispersal in the environment [4, 5].

When taken up into the human body, the reactivity of NMs depends on complex physicochemical properties such as size, agglomeration state, dissolution kinetics, capping agent, surface charge, and specific surface area [7–9]. The small size and remarkably high surface area of NPs enhance their interaction with biomolecules, biological membranes, cells or tissues. The high surface area of metal- and metal-oxide based NPs such as copper, zinc oxide, silver, manganese oxide, and cerium oxide increases the potential that metal ions are released from these NPs as they dissolve [10, 11]. The so-called “Trojan-horse” mechanism, through which Ag NPs act as a vehicle that carries silver across the cell membrane followed by intra-cellular dissolution of Ag NPs to release Ag ions, has been proposed as one mechanism of Ag NP toxicity [12–14]. In the presence of molecular oxygen and protons, silver atoms on the surface of Ag NPs (Ag^0^) can be oxidized resulting in the release of Ag ions [15, 16]. Dissolution of Ag NPs to form Ag ions may lead to the formation of hydroxyl radicals [17]. The formation of hydroxyl radicals can go through a process similar to the Fenton reaction, in which Ag NPs act as a Fenton-like reagent reaction [Ag + H_2_O_2_ + H^+^  = Ag^+^  + •OH + H_2_O] [18, 19], thus contributing to the formation of reactive oxygen species (ROS). Still, Ag ion release cannot alone account for the observed toxic effects [20]. The toxicity mechanism for Ag NP could be related to their small size, the amount of released silver ions, or a combination of both [13, 21].

Released ions from Ag NPs bind to ligands, creating a mixture of metal ion-ligand complexes. Silver is a soft acid according to the Pearson (HSAB) acid–base theory, therefore it shows high affinity for soft bases, and among them, it is particularly affine for thiols [10, 22, 23]. Thiol groups of cysteine residues are crucial for many proteins to maintain their integrity and function [24, 25]. The most abundant thiolated molecules in human cells include some proteins, especially metallothioneins and Zn-finger proteins, and small molecules such as glutathione (GSH) [26–28]. These molecules play an important role in maintaining the oxidative balance [26], metal homeostasis and DNA integrity in cells [29]. Ag ions complexation with these thiol groups may induce protein misfolding, scavenge thiolated molecules, thereby hindering their function and impairing cellular antioxidant mechanisms [14, 30, 31]. Due to the complex nature of Ag NP exposure, there remains uncertainty and to some extent controversy, regarding the level to which each constituent —ion, ion-protein complex, particle— contributes to cellular toxicity.

Considering their presence in food additives, food packaging, in textiles such as clothing and bedding and in toothbrushes, hair straighteners, disinfectant sprays as cosmetic and hygiene products, the major routes of exposure of Ag NPs are dermal contact [32], ingestion [33], and inhalation [34]. According to European Commission Scientific Committees, the main target organs for Ag NP in the human body are the spleen, liver, and kidney, with less distribution to other organs [20]. In the aforementioned study from the European Commission Scientific Committees, tissue distribution of 20 nm, 80 nm, and 110 nm of Ag NPs were investigated in rats after single and repeated intravenous administration of 1 mL/animal (~ 25 µg/mL, approx. 0.1 mg/kg bw/d) by Lankveld et al. [35]. Following single exposure, highest silver concentrations per gram organ were found in spleen followed by liver for both 80 and 110 nm particles. In spleen, Ag NP concentrations were approxiately 20 fold lower for 20 nm particles compared to the larger particles. Concentration of 110 nm particles were found around 1600 ng per gram spleen. Ag NP concentrations in liver increased with particle size (169, 539 and 1077 ng/g liver for 20, 80 and 110 nm particles, respectively). In the other organs evaluated (kidney, heart, lungs, testes and brain) silver concentrations were much lower and comparable for all sizes [35].

There are studies that recorded high levels of silver accumulation in the brain and testicles [20, 33, 35–39], although the significance for toxicity is unknown [20]. In 2018, the Scientific Committee on Consumer Safety (SCCS) of the European Commission recommended to collect information on the reproductive toxicity of Ag NPs [40]. The SCCS also noted the lack of information on systemic availability via the relevant uptake route(s) that would allow drawing conclusions on reproductive system toxicity [40]. Therefore, the potential toxicity mechanism of Ag NPs is a matter of great concern with regards to reproductive toxicity [41].

Exposure to nanoparticles may cause adverse effects on the reproductive function and fertility in adult males, including impact on reproductive cells, spermatogenesis, the seminiferous tubules, and testes [42]. In vitro studies show that Ag NPs cause the alteration of germ cells and somatic cells function mainly due to cell membrane peroxidation, oxidative stress, mitochondrial damage, DNA damage, and apoptosis [43–45]. Necrotic spermatogonial cells, degenerative alterations in the cellular architecture of testes and epididymis are reported in in vivo models [46, 47]. In some animal models, the accumulation of NPs in the testes is demonstrated [48, 49]. It is also reported that Ag NPs affect reproductive hormone levels such as testosterone and androgen hormones [47], sperm quantity and quality [46], which suggest potential consequences for male fecundity.

Likewise, in non-mammalian models, Ag NPs reproductive toxicity has been extensively studied. Ag NP considerably decrease reproductive potential in *Caenorhabditis elegans* [50]. Yan et al. prove the maternal transfer of Ag NPs to offsprings in *Daphnia magna* together with inhibition of the reproduction capability of F_0_ and F_1_ generations [51].

To support effective risk assessments of chemicals, The Organisation for Economic Co-operation and Development (OECD) has introduced the Adverse Outcome Pathway (AOP) conceptual framework [52], which is designed to organize toxicological information, thereby assisting integrated approaches to testing and assessment strategies [52]. Considering the creation of non-animal testing approaches, emphasis has been placed on AOPs as a conceptual support for developing in vitro and in silico testing strategies [52]. This framework presents a sequence of events commencing with initial interaction(s) of a stressor, which defines the perturbation in a biological system (i.e., molecular initiating event, MIE), and then a dependent series of intermediate key events (KEs), ending up in an adverse outcome (AO) [52, 53]. KEs describe a toxicological response and are linked to one another by a Key Event Relationship (KER), which establishes one KE as upstream and one KE as downstream [52, 53]. The AOP-Wiki database [54] serves as the primary repository of qualitative information for the international AOP development effort.

Within AOP-Wiki, one AOP that describes reproductive failure due to Ag NP exposure is available, which is AOP207 [55]. It has been established on the non-mammalian model *Caenorhabditis elegans (C. elegans)* by using a Bayesian network (BN) model [56]. The MIE of this AOP is oxidative stress through NADPH oxidase activity, reproduction failure is the outcome. PMK-1 (P38 MAPK) activation, HIF-1 activation, mitochondrial damage, DNA damage, and apoptosis are the described key events. Another AOP regarding reproductive toxicity of Ag NPs has been reported in zebrafish [57]. It describes that Ag NPs exposure causes oxidative stress, induces germ cells apoptosis via mitochondrial-dependent pathway, and ultimately impairs the reproduction in zebrafish [57]. Moreover, some reproductive failure-releated AOPs exist for other NPs such as graphene oxide and UV-activated Titanium dioxide NPs (TiO_2_ NPs). First, in AOP210 [58] the comprehensive mechanism of stress response to graphene oxide NPs is investigated in *C. elegans* using transcriptomics, metabolomics and lipidomics. Based on the results, the authors propose an AOP for oxidative stress leading to reproductive failure in *C. elegans*. This AOP includes the KEs increased oxidative stress, activation of c-Jun N-terminal kinase (JNK) and activation of transcription factor DAF-16/FOXO, inhibition of WNT signaling, defect embryogenesis, with the AO being reproductive. In addition, in AOP208 [59], JAK/STAT and TGF-beta pathway activation leading the reproductive failure has been used to describe the mechanism by which UV-activated TiO_2_ NPs affect the reproductive function [60].

Anticipating Safety Issues at the Design Stage of NAno Product Development (ASINA) EU Horizon 2020 (H2020) project is developing a specific Safe by Design (SbD) Management Methodology, consistent with modern business management systems, to deliver SbD solutions and inform design decisions. Within this project, the investigation of toxicity mechanisms of antimicrobial NMs that born to exert a toxicity effect is pivotal to support their design and match safety requirements. The current literature is reviewed in order to identify major areas of concerns related to the toxicity of the NM categories under investigation, using an AOP-oriented approach. With this perspective, in the present study, the reproductive toxicity of Ag NPs toward male mammalian models has been reviewed and a putative testable AOP is proposed, since previous related work has only focused on non-mammalian models. Based on the existing literature, we evaluated the MIE, KEs and AOs related to the induction of reproductive failure. As a follow-up of the present investigation, we expect to compare the AOPs of different models, including identified toxicity thresholds, so as to propose the most promising design options (concentration, physicochemical features, stabilizing agents) able to guarantee a safe use of Ag NMs.

## Methods

### Literature search

**Fig. 1:**
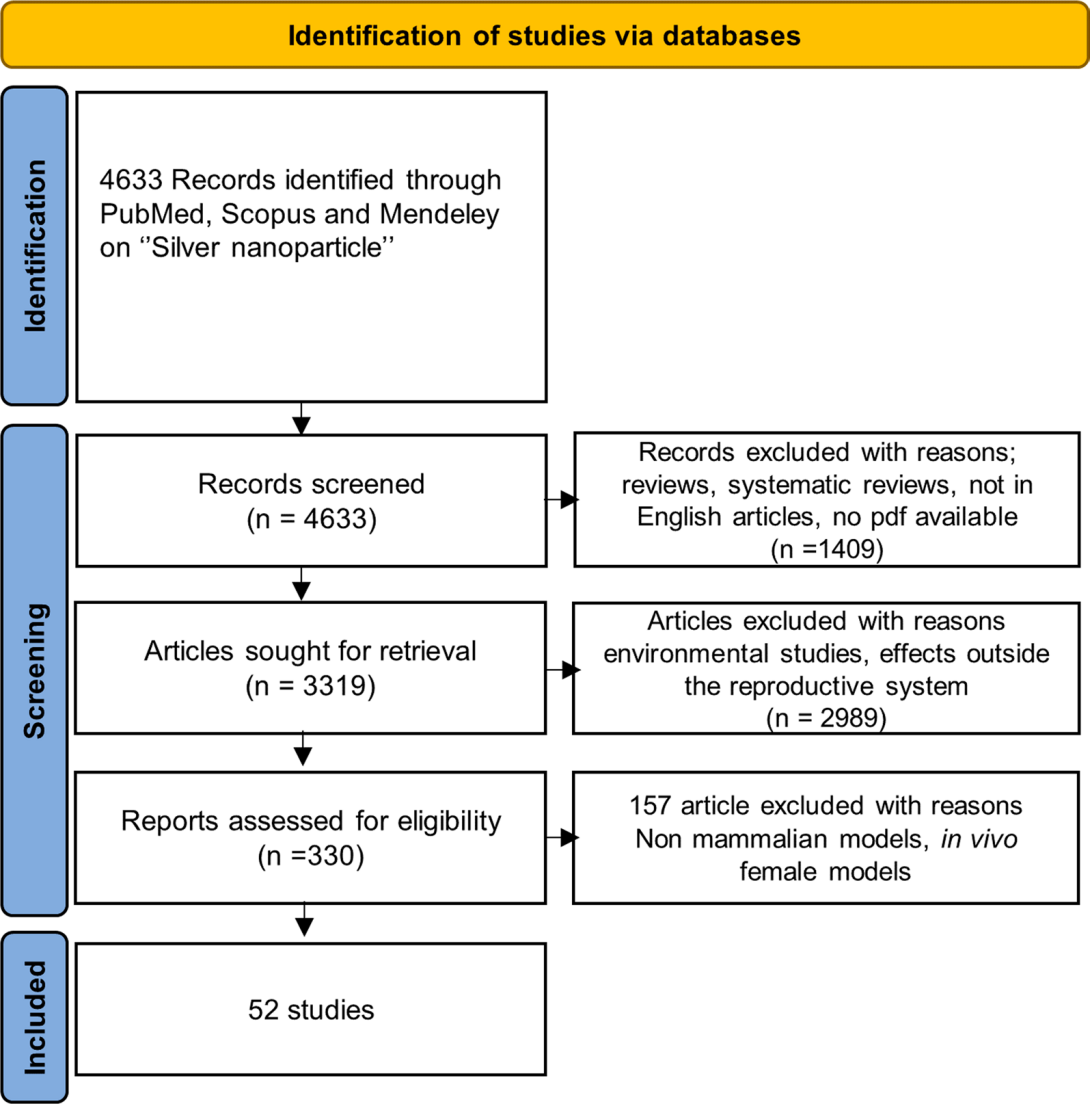
Scheme of evaluation of the literature between 2007 and 2022

The searches for peer-reviewed research publications on the toxicity of Ag NPs were conducted in PubMed and Scopus and limited between 2007 and 2022 according to the Preferred Reporting Items for Systematic Reviews and Meta-Analyses (PRISMA) recommendations [61]. The used search string was: (("silver"[MeSH Terms] OR "silver"[All Fields]) AND ("nanoparticles"[MeSH Terms] OR "nanoparticles"[All Fields]) AND ("toxic"[All Fields] OR "toxical"[All Fields] OR "toxically"[All Fields] OR "toxicant"[All Fields] OR "toxicities"[All Fields] OR "toxicity"[MeSH Subheading] OR "toxicity"[All Fields] OR "toxics"[All Fields]). In Scopus, the used keywords were: (silver AND nanoparticle AND toxicity). As shown in Fig. 1, a total of 4633 articles were identified. Among them, the articles not written in English, those that could not be accessed because they were either non-open access or because the main authors did not answer to our pdf request, as well as systematic reviews were excluded and this resulted in 3319 articles. Then, articles related to plant species or target organs outside of the reproductive system were eliminated. Finally, 330 articles were found to be related to reproductive toxicity of Ag NPs. Articles reporting data collected on in vivo female models, non-mammalian models such as drosophila, medaka, nematodes and zebrafish were further excluded. Finally, 52 articles were selected to identify how Ag NPs affect reproductive function in male in vivo and in vitro models.

The articles were separated into two parts, in vivo and in vitro. While 44 studies were in vivo studies, 6 of them were in vitro studies. Two study comprises both in vivo and in vitro models; therefore, they were evaluated in both groups for the corresponding parts. That is, 46 in vivo data and *8 *in vitro data were collected from 52 studies in total.

### Quality assessment

Study quality, study design adequacy for their intended measures and the reliability of outcome were assessed in these 52 studies by using the Toxicological data Reliability Assessment Tool (ToxRTool) [62]. These studies were evaluated in terms of the nanoparticle identification (synthesis, characterization, p-chem informations) (group I), in vivo or in vitro test system characterization (animal source, age, body weight information, housing-feeding conditions, cell types, cultivation conditions) (group II), study design description (administration route, dose, frequency/duration of exposure etc.) (group III), study results documentation (endpoints, methods judgment) (group IV), and plausibility of study design and results (group V).

The studies were scored between 0 and 21 points or 0–18 points for in vivo and in vitro, respectively (reliable without restrictions, in vivo: 18–21 points, in vitro: 15–18 points; reliable with restrictions, in vivo: 13–17 points, in vitro: 11–14 points; not reliable, in vivo: < 13 points, in vitro < 11 points). Studies with a score of ≥ 13 (in vivo) and ≥ 11 (in vitro) points were classified as high quality.

### Database analysis to identify potential KEs

The selected high quality studies were analyzed and reported in detail in our database as recommended by AOP-expert groups [53, 63, 64]. The physicochemical properties of the nanoparticles used in these articles (particle size, shape, surface area, surface charge, agglomeration status, surface coating, purity), characterization of the test organism used (animal sex, age, weight, cell type), route of administration, exposure dose and duration, methods, biological endpoints, and key results were reported separately for each article. The aim of this evaluation was to understand connection between p-chem properties and relevant key events and adverse outcomes, which would feed the ASINA SbD strategy.

Biological endpoints, measurements, methods were identified to define the standard strategy for nanoparticle reproductive toxicity evaluation and are listed in Table 1. The terminology used in the title of KEs were chosen according to the OECD guideline on AOP development [52]. All KEs were evaluated for three main criteria: credibility, measurability, and regulatory compliance. After that, KEs were classified at each major level of biological organization (molecular, cellular, tissue, organ, individual) and selected with respect to their potency to be measured in a relatively routine manner, in opposition to those that require highly specialized expertise or equipment. To establish the AOP frame, KEs that were reported as either transient or reversible were avoided.

**Table 1:** Database summary of the analysis of 4 studies out of 48 selected studies

Ag NP p-chem	Experimental model and mode of exposure	Exposure dose, sampling time		Biological/toxicological Endpoints	Actual measurements and methods	Result	Potential key event(s) (KE nr. from AOP-Wiki)^a^	Potential adverse outcomes (AOs) associated with the key event (AO nr. from AOP-Wiki^a^	References
Size: 50 nm Shape: Spherical Hydrodynamic size:113.4 ± 12.1 nm Zeta potential: − 12.30 ± 0.4 mV	Adult male SD rats Oral gavage	50 mg/kg bw, 3 months		Sperm Evaluation	Sperm Motility, Concentration, and Viability by eosin staining	Increased sperm morphological abnormalities Decreased sperm concentrations motility and viability	Decreased sperm quantity or quality in the adult, Decreased fertility (ID505, ID520)	Impaired fertility (ID330, ID406)	[46]
				Oxidative Status	GSH level, CAT activity and lipid peroxidation MDA content in testicular tissue by commercial kits	Decreased CAT activity, Increased MDA content, Non-significant change in GSH level	Decreased protection against oxidative stress, Occurance oxidative stress (ID210, ID1112, ID1249, ID1538, ID1869) Lipid Peroxidation (ID1445 or ID1511)	Oxidative Damage (ID356)	
				Hormonal Assessment	Serum testosterone, LH and FSH by ELISA kit	Decreased testosterone, LH and FSH levels	Reduction, testosterone level (ID1613, ID1689, ID1612) Reduced, Gonadotropins (ID1986)	Decreased sperm quantity or quality in the adult, Decreased fertility (ID505, ID520)	
				DNA damage	DNA strand breaks in testicular tissue by COMET assay	Increased DNA damage	Increased DNA damage (ID1194)	DNA Damage (ID1194)	
				Histopathological Examination	Testis and Seminiferous tubules by Hematoxylin and Eosin (HE) staining	Testis and Seminiferous tubules Histological alterations Necrotic spermatogonial cells	Testicular atrophy (ID1506)	Male reproductive tract malformations (ID348) Reduced, Reproductive Success (ID675)	
Size:45 nm PVP (< 1%) Zeta potential: − 20 mV	New Zealand White male rabbits Intravenous	5 mM AgNP solution (0.6 mg/kg bw) 2 months (weekly)		Sperm Evaluation	Sperm Volume, Concentration, and Viability	Decreased sperm motility, concentration and volume	Decreased sperm quantity or quality in the adult, Decreased fertility (ID505, ID520)	Impaired fertility (ID330, ID406)	[88]
				Oxidative Status	MDA content, NO concentration, CAT and GPX Activity in sperm and blood samples by commercial kits	Increased NO, and MDA content Decreased CAT activity, and GSH level	Decreased protection against oxidative stress, Occurence oxidative stress (ID210, ID1112, ID1249, ID1538, ID1869) Lipid Peroxidation (ID1445 or ID1511)	Oxidative Damage (ID356)	
Size:40 nm Shape: Spherical	Adult male NMRI mice Oral gavage	500 mg/kg bw with a time interval of 24 h for 35 days		Oxidative Status	Total antioxidant capacity and Lipid peroxidation parameters	Decrease in the total antioxidant capacity, Increased MDA content	Decreased protection against oxidative stress, Occurence oxidative stress (ID210, ID1112, ID1249, ID1538, ID1869) Lipid Peroxidation (ID1445 or ID1511)	Oxidative Damage (ID356)	[98]
				Hormonal Assessment	Serum testosterone level by comercial kits	Decreased testosterone hormone	Reduction, testosterone level (ID1613, ID1689, ID1612)	Decreased sperm quantity or quality in the adult, Decreased fertility (ID505, ID520)	
				Histological parameters	Testis, volume of interstitial tissue and seminiferous tubules	Decreased mean volume of testicular tissue and volume of seminiferous tubules Decreased sperm density, mean number of spermatocytes, mean number of Sertoli cells	Testicular atrophy (ID1506)	Male reproductive tract malformations (ID348) Reduced, Reproductive Success (ID675)	
Size:100 nm Shape: Spherical SSA: 7.5329m^2^/g Zeta potential: − 18.9 mV	Male Rats Sub dermal	10 and 50 mg/kg bw, 7 and 28 days		Sperm Evaluation	Sperm motility, velocity by HE staining	Decreased sperm motility and velocity	Decreased sperm quantity or quality in the adult, Decreased fertility (ID505, ID520)	Impaired fertility	[47]
				Oxidative Status	MDA, GSH, CAT	Increased Lipid peroxidization, Decreased SOD, CAT, GSH and total thiols	Decreased protection against oxidative stress, Occurence oxidative stress (ID210, ID1112, ID1249, ID1538, ID1869)	Oxidative Damage (ID356)	
				Hormonal Assessment	Testosterone, LH and FSH	Decreased testosterone, LH and FSH levels (dose dependent	Reduction, testosterone level (ID1613, ID1689, ID1612) Reduced, Gonadotropins (ID1986)	Decreased sperm quantity or quality in the adult, Decreased fertility (ID505, ID520)	
				Histological parameters	Cellular achitecture of testes and epididymis	Degenerative alterations in the cellular architecture of testes and epididymis	Testicular atrophy (ID1506)	Male reproductive tract malformations (ID348) Reduced, Reproductive Success (ID675)	

^a^As several KEs and AOs are related to some of these cellular mechanisms, we indicate the title of only some of them, but the IDs of all of them are cited

## Results and discussion

### Quality assessment of the identified studies

The quality of 52 selected studies were assessed using ToxRTool. Four records were analysed as poor studies because of missing material characterization and/or usage of ultra-high concentrations or doses and/or no controls for biological endpoints have been included. Seven records were acceptable studies, with most quality criteria fulfilled but not strictly all. Finally, forty-one records were considered good studies, with all quality criteria fulfilled.

Overall, the 48 selected studies were classified as good and acceptable quality and these were later used in subsequent data evaluations.

### Dataset evaluation

Datasets were created through our database containing a summary of the selected in vitro and in vivo studies. The in vitro datasets comprised studies performed on germ cells, Leydig, Sertoli cells or human semen. In these studies, the used cells were either commercial cell lines including mouse Sertoli cells (TM4 and 15P-1) or Leydig cells (TM3), or primary Sertoli cells (collected from 50 to 54 weeks-old Cobb-500 roosters), or C18–4 mouse spermatogonial stem cells established from type A spermatogonia isolated from 6-day-old mouse testes.

Among the in vivo studies, 17 studies used the oral route of exposure, i.e. Ag NPs were administered via oral gavage or through food/water, 13 used intraperitoneal injection, 9 used intravenous injection, 1 used subdermal, 1 intratracheal, and 1 intratesticular administration routes, which were mostly conducted in rats [25 studies], mice [15 studies] and rabbits [2 studies].

The dose ranges used in these studies were quite wide, for example in the 17 oral administration studies, 0.015 to 500 mg/kg/bw doses were applied. The dose was generally linked with those selected in previous studies. Some of the studies pointed that the investigated doses were in the range between the lowest observable adverse effect level (LOAEL) and a no observable adverse effect level (NOAEL) [46, 65], i.e., 125 mg/kg and 30 mg/kg, respectively, as suggested by a 90-day oral toxicity study, based on signs of liver toxicity [66].

**Fig. 2:**
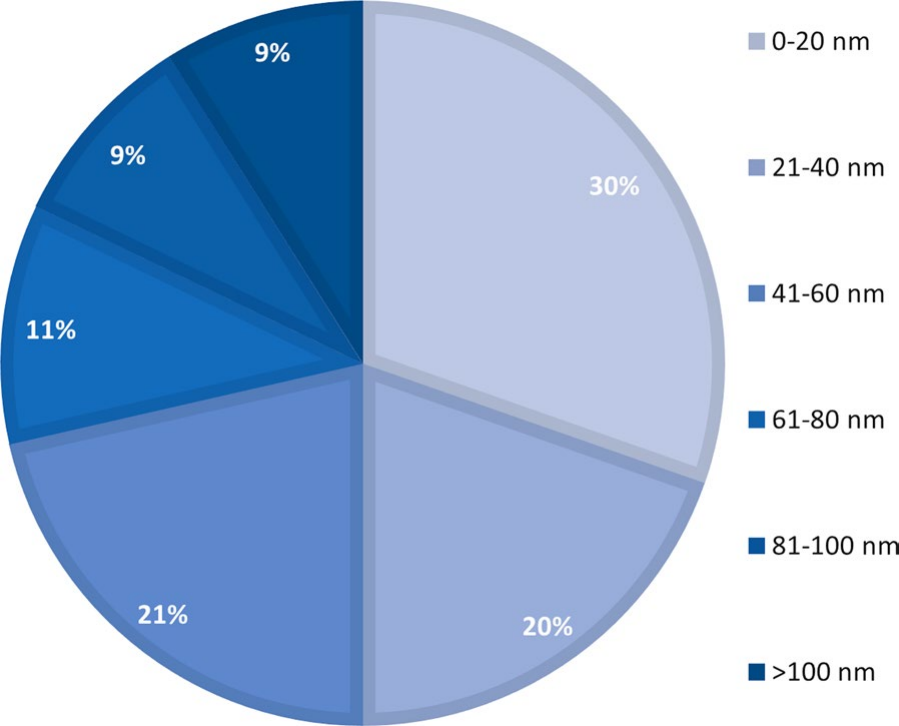
Size range of Ag NPs used for both in vivo and in vitro reproductive toxicity studies indicating the most prevalent size of Ag NPs (0–20 nm) employed for reproductive toxicity studies

In each study, the physicochemical properties of the used Ag NP was strictly reviewed, so that we could further identify the influence of these properties on the adverse outcome on the reproductive system. Ag NPs size range varied between 8.92 and 200 nm (Fig. 2).

Nine studies reported that the used Ag NPs were coated with polyvinyl pyrrolidone (PVP) or citrate. Moreover, some studies used Ag NPs produced via green method such as biogenic production in *Bacillus funiculus*, baker’s yeast *(Saccharomyces* cerevisiae) or *V. opulus* L. fruit extract. The studied Ag NPs were mostly spherically-shaped.

### Potential molecular initiating events

Two mechanisms involved in the toxicity of Ag NPs have been proposed. First, silver ions are formed and released, mediated by the oxidation of the metallic silver core, inducing the formation of ROS [67]. Second, silver ions interaction with enzymes and proteins containing thiol groups, such as metallothioneins or zinc-finger proteins, affect cellular processes such as cellular respiration and antioxidant defense system, possibly resulting in cell death [13, 14, 30, 68, 69]. Silver ions, indeed, are soft acid according to the HSAB principle, consequently they show high affinity towards soft bases and among them silver is highly affine for biomolecules containing thiols. However, it is important to go one-step back and explore the link between the oxidative stress and Ag NP dissolution to understand via which mechanism Ag NPs induce oxidative stress, in order to identify molecular initiating event(s).

Ag NP dissolution mechanisms are well described [10, 17, 70, 71]. The release of ions from Ag NPs has been shown to be an oxidation involving dissolved oxygen and protons. The reaction stoichiometry is as shown in the Eq. (1) [19].

1 $${2{Ag}^{0}}_{\left(s\right) }+ \frac{1}{2} {O}_{2(aq)}+2{H}_{(aq)}^{+}\leftrightarrow {2Ag}^{+}+ {H}_{2}O$$

Dissolution of Ag NPs to form Ag ions may accompany the decomposition of hydrogen peroxide under acidic conditions leading to the formation of hydroxyl radicals [17]. The formation of hydroxyl radicals can go through a process similar to the Fenton reaction, in which Ag NPs act as a Fenton-like reagent [17]. Under neutral and alkaline conditions, the reaction of Ag NPs with H_2_O_2_ generates oxygen instead of °OH radical [19]. The pH of the environment plays a role in the Ag NPs dissolution, as illustrated by Eq. (2) [71].

2 $${Ag}^{0}+ {H}_{2}{O}_{2}+ {H}^{+ }\to {Ag}^{+}+ {}{\cdot}\,OH+{H}_{2}O$$

The ability of generating radicals has been reported across a variety of metal and metal oxide NPs, such as copper nanoparticles, zinc oxide nanoparticles, Ag NPs [72–74]. Previous reports using electron spin resonance (ESR) coupled with spin trapping and spin labeling prove that free radicals are derived from the surface of Ag NPs [75, 76].

Inorganic NPs preferably enter into the cells via endocytosis [77]. Endocytosis can be categorized into phagocytosis and pinocytosis [78]. The uptake of larger particles (> 500 nm) is usually associated with phagocytosis. Pinocytosis can be classified into clathrin mediated endocytosis (CME) and caveolae mediated endocytosis (79). The internalization of nanoparticles with particles size < 200 nm usually proceeds by CME. Smaller nanoparticles (< 50 nm) undergo caveolae mediated endocytosis [80]. The uptake of NPs can be influenced by factors such as the physicochemical properties of NPs and cell types [81]. In cells exposed to AgNPs, the NPs are observed in early endosomes originating from membrane invagination [82, 83], which then fuse into late endosomes and ultimately to lysosomes. It is also supposed that AgNPs may directly cross the membrane to reach the cytoplasm, possibly by direct membrane translocation (81). The exact mechanisms mediating the penetration of AgNPs through the membrane still remain to be elucidated. Whatever the route of internalization, the local environment around Ag NPs drastically changes. In the favor of low lysosomal pH, Ag NPs undergo intracellular dissolution, leading to Ag(I) species [12, 22]. This behavior is related to the so-called “Trojan horse” mechanism and leads to high Ag(I) concentrations in cellular compartments that Ag ions would otherwise not reach. Intracellular Ag(I) is a chemically reactive form of silver and shows remarkable affinity to zinc-finger domains of proteins, thiol-containing enzymes and molecules, mainly GSH and metallothioneins. Binding to these ligands leads to the subsequent formation of intracellular Ag(I)-thiolate complexes [30, 84, 85]. Such interaction affects the native domain structure of these proteins, which plays a role in maintaining the cellular homeostasis and antioxidant systems. As a consequence, it will influence their biological functions [14, 69, 84–86].

Due to the abovementioned mechanisms, since a MIE describes an initial point of interaction between stressors and the biomolecule, we propose that the impairment of intracellular SH-containing biomolecules can be defined as a MIE of the pAOP described here. Indeed, the release of Ag(I) in solution, the consequent production of ROS together with the thiol-Ag ion complexation would activate such MIE and lead to a chain of intracellular consequences ultimately leading to reproductive toxicity.

In our MIE evaluations, we reviewed AOP207 [55], examining the reproductive toxicity of Ag NPs in worms. This AOP focuses on identifying potential MIEs on Ag NPs induced reproductive toxicity in *C. elegans* (Table 2) and is still under development. The authors examine the question of how Ag NPs cause ROS production in *C. elegans*. They state that ROS can be formed on the surface of nanomaterials or that following the NP internalization endosomes are formed and ROS are produced by NADPH oxidase. They examine whether ROS arise directly from Ag NPs or indirectly through the action of NADPH oxidase. Finally, they identify NADPH oxidase as MIE, and reproduction failure as the outcome in *C. elegans*. However, any general correlation between the findings from *C. elegans* and in vitro and in vivo mammalian studies on the toxicity of Ag NPs is lacking [87].

**Table 2:** Male reproductive system AOPs on AOP-Wiki

AOP ID:	Title	KE	AO	Taxonomic Applicability	References
18	PPARα activation in utero leading to impaired fertility in males	MIE: Activation, PPARα KE1: Decrease, Steroidogenic acute regulatory protein (STAR) KE2: Reduction, Cholesterol transport in mitochondria KE3: Reduction, Testosterone synthesis in Leydig cells KE4: Reduction, Testosterone level KE5: Decrease, Translocator protein (TSPO)	Impaired, Fertility Malformation, Male reproductive tract	*Rattus norvegicus* *Homo sapiens* *Mus musculus*	[138]
64	Glucocorticoid Receptor (GR) Mediated Adult Leydig Cell Dysfunction Leading to Decreased Male Fertility	MIE: Glucocorticoid Receptor Agonist, Activation KE1: Repressed expression of steroidogenic enzymes KE2: Increased apoptosis, decreased number of adult Leydig Cells KE3: Reduction, Testosterone synthesis in Leydig cells KE4: Reduction, testosterone level KE5: Decreased sperm quantity or quality in the adult, Decreased fertility	Impaired, Fertility	*Rattus norvegicus*	[151]
207	NADPH oxidase and P38 MAPK activation leading to reproductive failure in Caenorhabditis elegans	MIE: Activation, NADPH Oxidase KE1: ROS formation KE2: Increase, Oxidative Stress/Activation, PMK-1 P38 MAPK KE3: Activation, HIF-1 KE4: Increased, DNA Damage-Repair KE5: Damaging, Mitochondria KE6: Apoptosis	Reproductive failure	*Caenorhabditis elegans*	[55]
208	Janus kinase (JAK)/Signal transducer and activator of transcription (STAT) and Transforming growth factor (TGF)-beta pathways activation leading to reproductive failure	KE1: Activation, JAK/STAT pathway KE2: Activation, TGF-beta pathway	Reproductive failure	*Caenorhabditis elegans*	[59]
322	Alkylation of DNA leading to reduced sperm count	MIE: Alkylation, DNA KE1: Inadequate DNA repair KE2: Increase, DNA strand breaks KE3: Increase, Apoptosis	Reduce, Sperm count	No information	[122]
323	PPAR alpha Agonism Impairs Fish Reproduction	MIE: Activation, PPARα KE1: Decreased, cholesterol KE2: Decreased, 11KT KE3: Impaired, Spermatogenesis KE4: impaired, Fertility	No information	Teleost fish	[152]
444	Ionizing radiation leads to reduced reproduction in Eisenia fetida via reduced spermatogenesis and cocoon hatchability	MIE: Deposition of Energy MIE: Increase in reactive oxygen and nitrogen species (RONS) MIE: Increase, DNA damage KE1: Increased, Oxidative Stress KE2: Increase, Apoptosis KE3: Decreased spermatogenesis KE4: Decrease, Fecundity KE5: Decrease, Reproduction	Decrease, Population growth rate	No information	[153]

### Identification and selection of key events

AOP-Wiki was screened to identify already-existing KEs that could describe the biological events reported in the 48 selected studies. The result of this analysis and screening is reported in Additional file 1, an extract of which, reduced to the analysis of 4 articles, is presented in Table 1.

Disruption of SH-containing molecules (MIE), such as glutathione, can cause oxidative stress through disruption of the antioxidant system, as described in the section above. In our database, the most reported biological events is Ag NPs exposure triggering oxidative stress, which is described in 22 out of the 48 articles at both cellular and tissue level. It has been shown that accumulation of Ag NPs led to cell depletion from the molecular antioxidant GSH [47, 88–90], and decreased super oxide dismutase (SOD) and catalase (CAT) activities [38, 47, 90–92], altered enzymatic oxidative defense system in male reproductive system [38, 46, 47, 90, 93, 94] and lead to increased ROS levels in human sperm [95], in mouse Sertoli cells (15P-1) [96], in somatic Leydig (TM3) and Sertoli (TM4) cells [44, 97] which eventually caused oxidative stress.

In addition, mitochondrial damage due to the impairment of metallothioneins (MIE) would result in oxidative stress by inhibition of electron transfer chain enzymes and perturbation of antioxidant system. Thus, it would increase mitochondrial ROS production, which may lead to mitochondrial damage including damage to respiratory chain and its membrane permeability. It has been shown that Ag NPs within the intracellular space has the potential to cause mitochondrial dysfunction by the depolarization of the mitochondrial membrane [97, 99]. Wang et al. reported damaged mitochondria in the testis upon Ag NP exposure to Balb/c mice [100]. These findings support the hypothesis in the biological plausibility perspective that Ag NPs interact with the thiol groups of the biomolecules, causing disruptions in the antioxidant system and thus triggering oxidative stress.

**Fig. 3:**
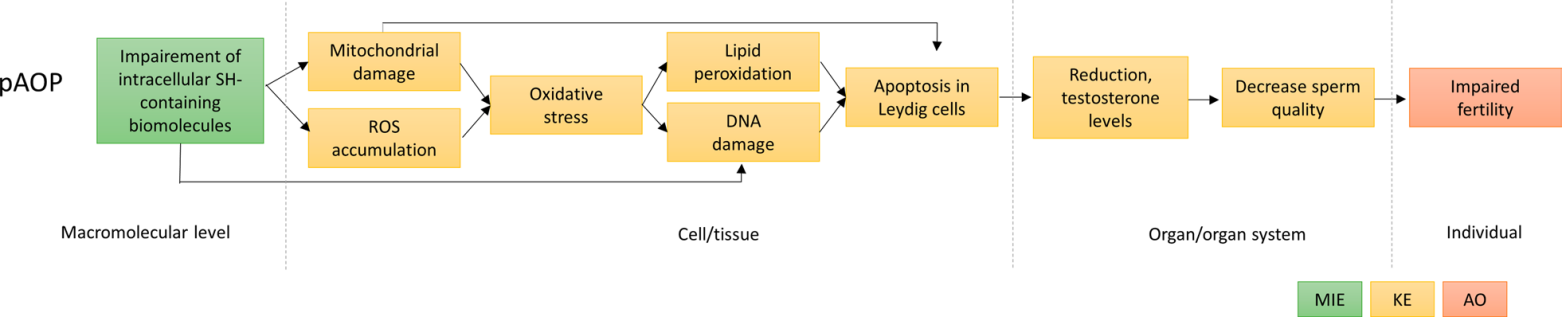
Proposed putative AOP: impairment of intracellular SH-containing biomolecules leading to impaired fertility

In addition to outlining the evidence supported by biological plausibility, there is also empirical evidence supporting this association in AOP-Wiki. AOP17 [101] has proposed a MIE similar to ours as the binding of electrophilic chemicals to SH(thiol)-group of proteins and/or to seleno-proteins, generating neurotoxicity. It is stated in AOP17 that soft metals like mercury binding to thiol/sulfhydryl/SH/SeH-groups results in structural modifications affecting the catalytic capacity of enzymes, and thereby reducing their capacity to neutralize ROS [102]. The relationship of this MIE and oxidative stress is classified as moderate in quantitative manner. The same could occur with Ag ions. Therefore, it can be assumed that impairment of SH-containing molecules like glutathione and metallothioneins can lead to both mitochondrial damage (KE1a) and ROS production (KE1b), eventually resulting in oxidative stress (KE2) as shown in Fig. 3.

Lipid peroxidation following Ag NP exposure has also been identified in our database. Lipid peroxidation byproducts including malondialdehyde (MDA), and thiobarbituric acid reactive substances (TBARS) have been shown to be significantly increased in serum, testicular tissue or in reproductive cells exposed to Ag NPs [46, 96, 98, 103, 104]. ROS-mediated lipid peroxidation is shown in at least two studies out of the 48 studies [38, 88]. Collodel et al. [88] confirmed the correlation between excessive radical generation, lipid peroxidation, and damage to the sperm membrane. Evidence supporting the KER between oxidative stress and lipid peroxidation was also provided by AOP-Wiki and the relation (KER ID:1727) is classified as high by weight of evidence and quantitative understanding. Therefore, it can be postulated that oxidative stress leads to lipid peroxidation, as reported in Fig. 3.

Ag NPs induced DNA damage is reported in some of the evaluated studies in in vitro germ cells, somatic cells [95, 105] and in in vivo sperm samples and testicular tissues [46, 89, 92, 93, 106–109]. It is well accepted that spermatozoa are extremely sensitive to the damaging effects of ROS due to the polyunsaturated fatty acids (PUFA) in their cell membranes and due to the lack of adequate DNA repair mechanisms [41, 110]. Reactive oxygen species induce intracellular oxidative burden by fostering the peroxidation of lipids. The sequence of events involves lipid peroxidation, loss of membrane integrity with increased permeability, reduced sperm motility, structural DNA damage and apoptosis [41]*.* According to AshaRani et al. and Carlson et al., ROS formation/oxidative stress was suggested to be a key event in DNA damage induction [99, 111].

Apoptosis is a widely observed response to Ag NP exposure, which is frequently reported through measurements of apoptosis-related proteins at the cellular level, or the tissue level with histopathological observations. At the cellular level, Ag NPs induce apoptosis in the mouse germ cell line C18-4 [112], in the mouse male-derived Sertoli cell line TM4 [97]. It is also suggested that accumulated ROS lead to apoptosis as downstream event in somatic Leydig and Sertoli cells [44]. Ag NPs induce expression of autophagy-related genes and activate signaling molecules involved in apoptosis [44]. Ntera2 cells (NT2, human testicular embryonic carcinoma cell line) are affected by Ag NPs which cause DNA strand breaks, reduce the cell proliferation and trigger apoptosis and necrosis [107].

Moreover, several in vivo studies demonstrate alterations in apoptosis-related gene expressions, increased ratio of Bax/Bcl-2 expressions (90, 93, 100, 113, 114) and mitochondria-dependent intrinsic apoptotic pathway in testes [93, 113]. An extensive gene expression analysis conducted on 383 genes by microarray shows great changes in apoptosis-related genes and proteins (caspase3 and Myc). This analysis shows apoptosis-related changes of testis morphology and sperm production, with the evidence of apoptotic nuclei in spermatogonia and spermatocytes in the testis [93, 100].

The histopathology assessment of tubular cross-sections of seminiferous tubules provides evidence of increased number of apoptotic germ cells such as spermatogonia, spermatocytes and spermatids and somatic Leydig cells.

[39, 115]. Testicular sections in rats treated with Ag NPs show decrease and disturbance in the spermatogenic cells arrangements, atrophied seminiferous tubules with degenerative Sertoli cell, and depletion in Leydig cells [89]. In addition, other studies using different target systems such as liver [31], colon [116], and endothelial cells [117] show that Ag NPs cause apoptosis in a p53-dependent process involving ROS and the c-Jun N-terminal kinase cascade, or via the IKK/NF-κB pathway. These results suggest the appropriateness of the KE ‘apoptosis’. Moreover, it is widely recognized that if cells fail to handle oxidative stress, then apoptosis will be triggered through downstream signaling pathways [118–121]. Therefore, we chose to define apoptosis as a downstream event of mitochondrial damage, DNA damage and lipid peroxidation in the pAOP that we propose (KE4).

**Fig. 4:**
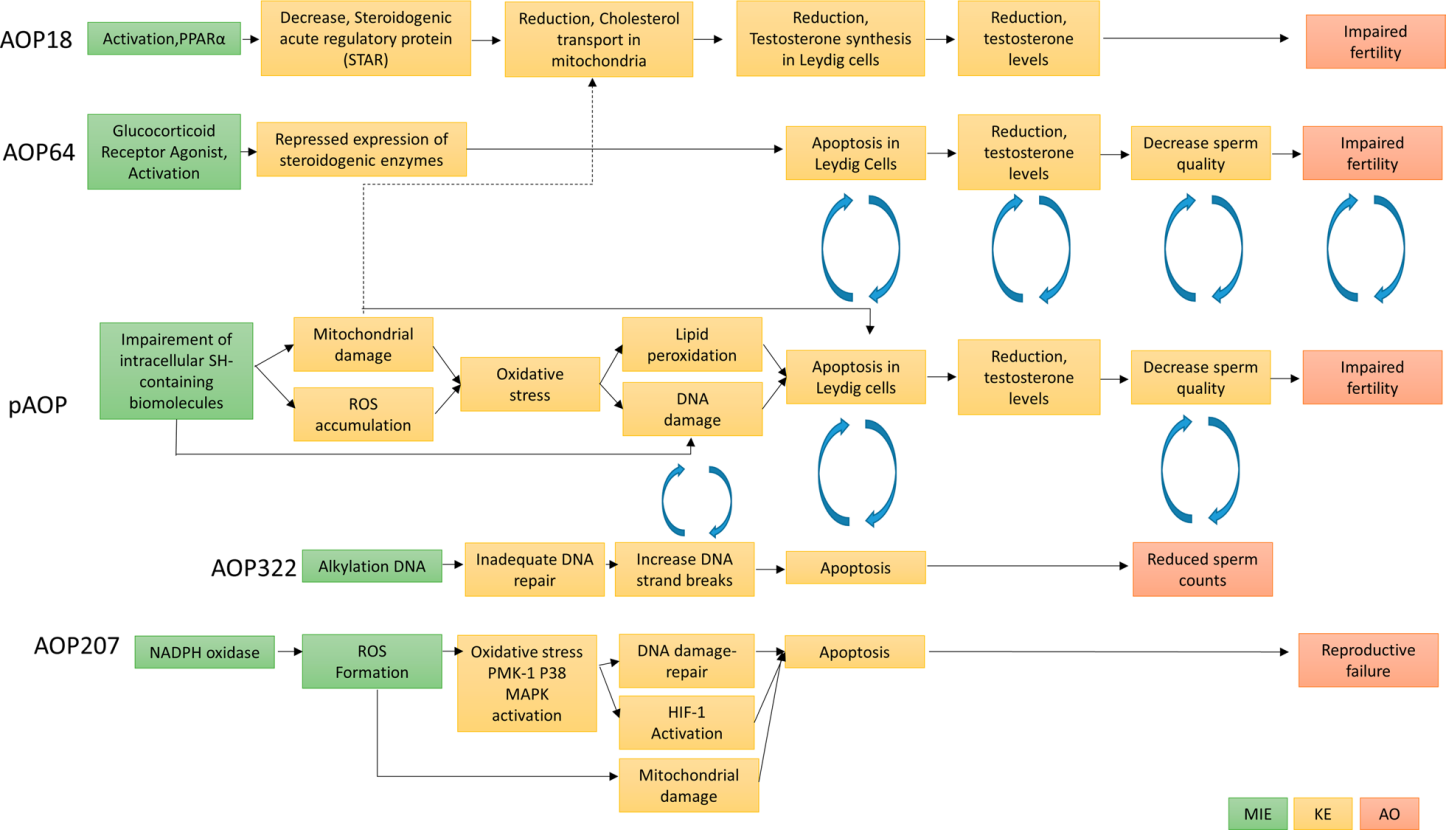
Putative AOP (pAOP) on Ag NPs reproductive toxicity relations with already existing AOPs, AOP18 [138] PPARα activation in utero leading to impaired fertility in males**,** AOP64 [151] Glucocorticoid Receptor (GR) Mediated Adult Leydig Cell Dysfunction Leading to Decreased Male Fertility, AOP207 [55] NADPH oxidase and P38 MAPK activation leading to reproductive failure in *Caenorhabditis elegans*

In AOP-Wiki, apoptosis, DNA damage, and sperm count relation was evaluated in AOP 322 [122] (Fig. 4). In this network, DNA alkylation (MIE) cause subsequent KEs as inadequate DNA repair, increased DNA strand breaks, increased apoptosis, and reduced sperm counts (Table 2). This AOP 322 is still under development, however, it provides a key sub-network that is possibly relevant to our pAOPs (Fig. 4). The AOP examining the reproductive toxicity of Ag NPs in worms [55] classified PMK-1 (P38 MAPK) activation, HIF-1 activation, mitochondrial damage, DNA damage, and apoptosis as KEs (Fig. 4). The authors performed correlation analysis between each KE on their AOPs and it has been proved that there is significant positive correlations between the exposure concentration of Ag NPs, ROS formation, the expression of bli-3 (NADPH oxidase), and mitochondrial damage. The most significant negative correlations were observed between the concentration of Ag NPs, reproduction and DNA repair gene expressions *in C. elegans* [56]. Our in vivo and in vitro data analysis is in good agreement with this AOP framework developed by Jeong et al., which has similar KEs as in our proposed pAOP on mammalian models.

Significant alterations of serum and intratesticular testosterone levels was observed upon Ag NPs exposure, as reported in a number of studies from our database [46, 47, 91, 92, 114, 123]. According to Attia et al. the significant decrease in the level of serum testosterone could be related with the adverse effects of Ag NPs in Leydig cells [91]. Circulating testosterone levels depend on the steroidogenic capacity of individual Leydig cells and the total number of Leydig cells per testis [124]. Leydig cell apoptosis causes the decrease in their number in the testis, which in turn affects testosterone level as shown in some studies [39, 125, 126] and further impact the spermatogenesis [127]. Therefore, the relationship between apoptosis of Leydig cells and alterations of serum and intratesticular testosterone levels is consistent with established biological knowledge.

On the other hand, there are studies showing that low testosterone levels are associated with impaired cholesterol transport in damaged mitochondria of Leydig cells [128, 129]. Mitochondrial steroidogenic acute regulatory protein (StAR) or translocator protein (TSPO) are responsible for cholesterol transport from the outer to the inner mitochondrial membrane [130]. In the inner membrane, cholesterol is converted into the Pregnenolone by CYP11a1 [130]. Afterwards, 3β-Hydroxysteroid dehydorgenase (Hsd3b), 17β-Hydroxysteroid dehydorgenase (Hsd17b), and CYP17A1 transform pregnenolone to testosterone [125, 130]. In our database, steroidogenesis perturbation by Ag NPs were shown in some reproductive toxicology studies. Garcia et al. [131] reported no change in the expression level of StAR after Ag NP administration although they detected increased Cyp11a1 and Hsd3b1 expression levels in CD1 mice. Increases in these two enzymes involved in the steroid biosynthetic pathway were consistent with observed increases in serum and intratesticular testosterone in this study. By contrast, Dziendzikowska et al. [132] reported decreased expression level of StAR, Cyp11a1, Hsd3b1 and Hsd17b3 in Wistar rats treated with Ag NPs. They also found a decreased plasma and testicular testosterone concentration after Ag NP treatment, which was correlated with decreased expression level of the aforementioned proteins. Similar to these results, in an in vitro study, Zhang et al. [105] showed inhibited StAR, Hsd3b1, and Hsd17b transcription in TM3 cells. They stated that decreased expression of Hsd3b1, and Hsd17b may be partially due to the reduced levels of StAR, which negatively affected testosterone production in TM3 cells [44]. When no modulation of StAR mRNA expression is observed, the author’s hypothesis is that it might be due to post-translational modifications that regulate StAR activity [39]. In fact, regulation of StAR gene expression is a complex process involving the interaction of a number of post-transcriptional mechanisms that govern mRNA and protein expression, as well as multiple signaling pathways that coordinate the cooperation of various hormones and transcriptional machinery (133, 134). These post-translational modifications to StAR (reviewed in [134]), may serve to enhance its stability or its ability to interact with other proteins necessary for cholesterol transport. On the other hand, as suggested by Dziendzikowska et al. [135], impaired steroidogenesis is probably resulting from the interactions of Ag ions with the thiol groups present in the inner mitochondrial membrane [136, 137]. This concurs well with the MIE in the putative AOP that we propose.

In AOP-Wiki, impairment of steroidogenesis was investigated regarding its role in reproductive toxicity, and we identified AOP18 [138] that describes the AO impaired fertility following a MIE that is Peroxisome Proliferator-Activated Receptor alpha (PPARα) activation in Leydig cells [139]. The development of this AOP relies on evidence collected from rodent models and incorporates human mechanistic and epidemiological data. The pathway comprises the activation of PPARα, followed by the disruption of cholesterol transport in mitochondria, impairment of hormonal balance which leads to malformation of the reproductive tract in males. PPARα is a transcription factor belonging to the nuclear receptor family, which also contains steroid and thyroid hormone receptors [140]. PPARs play an essential role in the metabolic regulation of lipids, particularly cholesterol, linking lipid metabolism with effects on reproduction. The effects of PPARα action on the reproductive system, as reported in AOP18, stem from limited experimental data showing associations between activation of this receptor and disruption of steroidogenesis [141, 142]. However, the major uncertainty in this AOP is the functional relationship between PPARα activation (i.e., the MIE) and cholesterol transport reduction [143]. Data gaps is defined as lacking of complete/pathway driven studies to investigate the effects of PPARs and their role in male reproductive development. On the other hand*,* in their evidence assessment, the authors found a moderate relation between cholesterol transport in mitochondria and testosterone synthesis. It is stated that decreasing the amount of cholesterol inside the mitochondria (e.g., by decreasing the expression of enzymes like StAR or TSOP) will result in a diminished amount of substrate for hormone (testosterone) synthesis. These results offer compelling evidence for the alteration of testosterone level in our pAOP. Therefore, we can assume that apoptosis in Leydig cells or its mitochondrial damage may lead to the endpoint of testosterone level reduction that we define as KE5 in our pAOP.

In our database, after intravenous injection or oral route administration, Ag NPs are shown to accumulate in the testes and are found in spermatids and ejaculated sperms, which suggests the likelihood that Ag NPs could pass through the blood-testis barrier (BTB) and eventually could impair the endocrine and reproductive functions [33, 37, 48, 49, 66, 144–146]. Arisha et al. showed increased Ag NPs levels in testes, reduced expression of tight junction proteins (occludin, claudin-11, and tight junction protein 1) resulting in BTB permeability increase [113]. They correlated these results with significantly reduced mRNA expression of hypothalamic GnRH1, testicular AR, and serum levels of follicle-stimulating hormone (FSH), luteinizing hormone (LH) and testosterone concentrations. The authors conclude that decreased testosterone level, mainly due to unbalanced sexual hormones signaling and testicular damage, affects spermatogenesis. In a very recent study investigating steroidogenesis in rat hippocampus upon exposure to Ag NPs, the alterations in expression of Star, Hsd3b3, and Hsd17b1 genes, involved in steroid metabolism, is shown [135]. Any interference with the normal functioning of the hypothalamic-pituitary–testicular axis can lead to reduced fertility, and if this interference persists, infertility could develop. Although these events do not occur in the reproductive organ itself but rather in the associated endocrine system, they contribute to Ag-NP reproductive toxicity and are complementary to all the other tested endpoints. Still, we chose not to include them in the current pAOP, which is focused exclusively on the male reproductive organ.

Sperm characteristics have a great importance in the prediction of fertility. Twenty-four out of the 48 identified studies analyzed sperm parameters after exposure to Ag NPs, including sperm morphology, viability, motility, and DNA damage. Upon Ag NP exposure, abnormal sperm morphology, decreased sperm viability and motility, increased sperm DNA damage were reported in a number of studies [46, 88, 90, 92, 100, 103, 123, 147]. Gromadzka-Ostrowska et al. [148] observed a decrease in the number of epididymal spermatozoa after 28 days in Wistar rats treated with a single injection of 20 nm AgNPs. The production of sperm requires a complex interaction between Sertoli, Leydig and germ cells. Any defect of these cells may prevent normal sperm production. In fact, the epididymis is necessary for post-testicular sperm maturation as it provides the milieu required for spermatozoa to gain the ability for fertilization (149). During the transit through the epididymis segments, the sperm released from the lumen of seminiferous tubules undergoes maturation and acquires motility and the ability to fertilize oocytes [149]. Cavallin et al. [108] showed reduced sperm storage and reduced sperm transit time after Ag NPs exposure to rats. This is explained as altered serum concentrations of testosterone, which could stimulate the contraction of the smooth muscle of the epididymis; as a result these increased contractions could contribute to the observed decrease in sperm transit time (108). Moreover, studies mostly emphasize that inadequate hormonal level may provoke low sperm quality and quantity [150]. In the AOP-Wiki database, the reduction of testosterone level is defined as an upstream event of decreased sperm quality. Therefore, we assigned decreased sperm quality and quantity (KE6) as a downstream event of reduction, testosterone level (KE5). When no modulation of StAR mRNA expression is observed, the authors hypothesis is that it might be due to post-translational modifications that regulate StAR activity (41). At the organ level, in vivo histopathological analysis of testis was conducted in 27 studies out of 48. Testis index and histological structure of testicular tissues, morphology, seminiferous tubule area, circumference, diameter and tubular degeneration/atrophy were the most studied parameters. A prominent atrophy of seminiferous tubules, thinning of the tubule wall, disorganization and vacuolization of germinal epithelium, and loss of spermatogenic cells in testis tissue of rats and mice exposed to Ag NPs are reported [47, 90, 93, 97]. In the interstitial tissue, Leydig cells are highly affected, presenting disrupted plasma membrane on extensive areas, with loss of cell organelles [93]. Shehata et al. showed significant reduction in the area, circumference and mean diameter of seminiferous tubules in Ag NP exposed rats [46]. These results suggest that Ag NPs may affect the testicular structure and decrease reproductive success.

As a summary, impairment of intracellular SH-containing biomolecules may lead to mitochondrial damage and ROS accumulation (KE1) and lead to oxidative stress (KE2) which further provoke DNA damage and Lipid peroxidation (KE3). Intracellular perturbations may lead to apoptosis in Leydig cells. At the organ/organ system level, these perturbations result in altered testosterone level (KE5) and decreased sperm quality (KE6). All these biological events, which emerged from our literature analysis, lead to the pAOP framework shown in Fig. 3.

### Network of AOPs

Then, the pAOPs presented in Fig. 3 was used to tentatively build a network of AOPs for reproductive toxicity. MIEs and KEs involved in male reproduction impairment-related AOPs were extracted from AOP-Wiki and are listed in the Table 2.

Some AOPs listed in this table are already discussed in the previous section. We observed that these AOPs share at least one common KE with our pAOPs. For example, oxidative stress, apoptosis, reduction of testosterone levels, decreased sperm quality events are shared by AOP 64, 207, 444. In Fig. 4, we interconnected these events with our pAOPs. While individual AOPs are likely to be activated by a limited number of reprotoxic compounds, interconnected AOPs that are linked by common KEs of single AOPs are likely to represent more realistic descriptions of the complexity of disease pathophysiology [154].

The AOP network reported in Fig. 4 also shows the potential knowledge gaps in internal associations between KEs. Complete/pathway driven studies investigating the effects of impairment of SH-containing biomolecules and their role in male reproductive development are lacking. For establishing a solid quantitative linkage, mode of action framework analysis for reproductive toxicity is needed. This figure also could serve as a candidate list of MIE that could provide clues for experimental verifications for future studies.

### Experimental methods for assessing the KEs

As suggested by Halappanavar et al. [63], AOPs can be used as a tool in the design of testing strategies to support the safety assessment of nanomaterials. In this regard, our database identified the various in vitro endpoints, methods and assays used to measure the KEs in this pAOP (Table 3).

**Table 3:** Summary information of the biological endpoints measured in the reproductive system toxicology

KE	Biological events/measurement	Methods	References
Cell viability		MTT, MTS, LDH, CCK-8 assay	[96, 97, 105, 107, 112]
Oxidative stress	ROS Production	H2DCFDA	[95, 97, 105]
	Lipid peroxidation, MDA content In testicular tissue and cells	ELISA, Western Blot, qRT-PCR	[89, 90, 94, 96, 98, 109]
	Enzymatic/non-enzymatic antioxidants GPX, CAT, SOD, TBARS, TAOC, GSH in Sperm, Seminal Plasma, and Blood, serum, testicular tissue homogenate and in vitro cells	ELISA, Western Blot, qRT-PCR	[46, 88, 89, 96, 104, 155]
DNA damage	DNA Strand breaks, In cells and the testis tissue	Comet Assay, DNA microarray analysis, DNA fragmentation	[46, 88, 89, 96, 107, 109]
Mitochondrial damage	Cell metabolic activity	MTT assay	[112]
	Mitochondrial membrane potential	Quantitative method uses a tetraphenylphosphonium ion (TPP+)-sensitive electrode, or by fluorimetric methods	[97]
Apoptosis, in germ Sertoli and Leydig cell	P53, BAX, bcl-2 gene expressions or cell apoptosis	Western Blot, qPCR, TUNEL Assay Flow Cytometry	[90, 93, 97, 112, 113, 166]
Reproductive hormone levels	Hormonal Assessment Serum Testosterone, LH and FSH, *P450scc*, *StAR*, Hsd3b, and *Cyp17a1* gene expression	Radioimmunoassay [167], ELISA, qRT-PCR, chemiluminescent protein immunoassay	[46, 89, 91, 98, 100, 132, 174]
Sperm evaluations	Morphology, Motility, Concentration, Count, Viability, Plasma membrane intergrity	Eosin/nigrosine staining, hemocytometer, Microscope	[46, 92, 95, 103, 146, 166, 175]
	Acrosome status	Fluorescence assessment (eg.chlortetracycline fluorescence assay)	[65, 146, 175]
	Sperm DNA integrity	Toluidine blue staining, Aniline blue staining, Acridine orange staining Eosin–nigrosine-staining	[38, 90, 103, 123, 148]
	Sperm DNA damage	Comet assay	[109, 148]
	The mitochondrial activity	Activity of cytochrome c oxidase	[65, 108]
	Spermatogenesis	The transcript expression of Gnrh1, Ar, Cyp11a1, Hsd3b1, Hsd17b3, Srd5a1, Cyp19a1, Star by qPCR Intratesticular steroid metabolism enzyme protein level aromatase (Aro) and 5α-reductase type 1 (Srd5a1) measurements	[108, 132]
Determination of silver concentration in organs		ICPMS, UV/vis proton spectrophotometery	[38, 114, 176]
Histopathological Examination	The seminiferous tubules area, circumference, and diameter, Testis index and histological structure of testicular tissues	Hematoxylin and Eosin light microscope Transmission electron microscopy (TEM)	[46, 100, 103, 123]

In studies examining oxidative stress, the intracellular level of ROS has often been evaluated using a fluorescent probe such as H2-DCF-DA [95, 97, 105]. MDA content in ELISA methods can be used to detect lipid peroxidation, which is one of the main indicators of oxidative stress [90, 94, 98]. With commercial kits, GSH levels and total antioxidant capacities can be measured, as well as analyzed at the level of antioxidant biomolecules and enzymes such as CAT, SOD [96, 104, 155]. GSH and GSSG levels can also be determined biochemically via high performance liquid chromatography (HPLC), capillary electrophoresis or microplates. Gene expressions of the antioxidant defense system can be measured by RT-qPCR. Mitochondrial dysfunction can be measured by colorimetric assays such as 3-(4,5-Dimethylthiazol-2-yl)-2,5-diphenyltetrazolium bromide (MTT), through assessment of mitochondrial membrane potential (MMP), mitochondrial ATP production, cytochrome c release, or mitochondrial DNA damage [97].

Although in the articles from our database DNA damage is evaluated using the comet assay, micronucleus assay and DNA fragmentation, a roadmap for testing DNA damage caused by nanomaterials was recently proposed by Elespuru et al. [156]. It includes the use of an in vitro gene mutation assay (OECD TG476 [157], HPRT or TG490 [158], mouse lymphoma TK ± assay) and a chromosomal damage assay (OECD TG487 [159] in vitro micronucleus assay or TG473 [160] chromosomal aberration assay). Eventually, optional assays are proposed, both in vitro (comet assay) and in vivo (comet assay, OECD TG489 [161]; transgenic rodent gene mutation assay TG488 [162]; erythrocyte micronucleus assay, TG474 [163]; bone marrow chromosomal aberration test, TG 475 [164]). Other non-guideline test methods to measure the DNA damage also exist although they are not discussed in the roadmap by Elespuru et al. [156]. For instance the detection of DNA repair proteins such as H2AX, 53BP1 or XRCC2 can be used, or high performance liquid chromatography (HPLC) coupled to tandem mass spectrometry (MS/MS) that quantifies very low levels of oxidative lesions to DNA [165].

In articles in our database, apoptosis was evaluated by western blot, qPCR, TUNEL Assay or Flow Cytometry [93, 97, 112, 166]. Other methods can also be used, including annexin V-FITC probes, with analysis of the relative percentage of Annexin V-FITC-positive/PI-negative cells by flow cytometry. The alteration of procaspases 7 and 3, Caspase-3 and caspase-9 activity, as well as the cleavage of the poly(ADP-ribose) polymerase (PARP) can be determined by western blotting or RT-PCR.

The OECD TG 456 [167] is a validated test guideline for in vitro screening of the effect of chemicals on steroidogenesis, specifically the production of 17ß-estradiol (E2) and testosterone. In vitro testosterone synthesis in Leydig cells can be measured by *P450scc*, *StAR*, Hsd*3b*, and *Cyp17a1* gene expression or indirectly by testosterone radioimmunoassay or analytical methods such as LC–MS or by isotope-dilution gas chromatography–mass spectrometry in serum [168, 169]. Sperm assessment includes the evaluation of sperm count and concentration (hemocytometer, automated image-based system), morphology and motility (microscope, automated image-based system) and viability (for example propidium iodide staining of necrotic cells, TUNEL assay staining apoptotic cells). Sperm DNA damage can be evaluated by Acridine orange assay. The principle of the assay is sperm DNA binds to the AO dye by acid denaturation. AO binding to intact DNA is visualized as green and damaged DNA as red by a microscope or flow cytometer [170]*.* Models that can be used as alternatives to animal experimentation for assessing this putative AOP on male reproductive function, i.e., in vitro models of Leydig cells, Sertoli cells, Sertoli-germ cell cocultures, as well as methods to prepare testicular organ and tissue culture systems can be found in the Database Service on Alternative Methods to animal experimentation (DB-ALM) [171]. Data generated by alternative methods or in vivo testing can be integrated in quantitative AOPs and can be validated for future studies.

Another purpose of our research in developing this pAOP was to observe how physicochemical properties of NPs influence the key events and their relationships. From the selected articles that were analysed, we identified Ag NP size, agglomeration state, surface coating and tendency to dissolve as key physico-chemical parameters that could influence their toxicity (Additional file 1). However, it is difficult to reach a definite conclusion, as the physicochemical characterization of Ag NPs in the considered articles is too diverse and sometimes lacks precision. It seems clear from these studies that controlling ion release could diminish the hazard potential of Ag NPs with respect to SbD approaches [22]. Therefore, it is highly recommended to analyze Ag ion release systematically in the published articles. Since ion release is higher when the nanoparticle is smaller and when the nanoparticle surface is uncoated or coated with a ligand that tends to desorb, we consider that both the size and surface coating are important parameters that influence Ag NP hazard potential, as previously suggested in studies related to other organs [14, 172, 173].

## Conclusion

This review discusses the effects of Ag NPs on male reproductive system in the concept of AOPs. By reviewing the existing literature, a putative AOP framework was constructed, where the MIE is identified as the impairment of intracellular SH-containing biomolecules, with subsequent key events that are mitochondrial damage, ROS accumulation, DNA damage and lipid peroxidation, apoptosis, reduction of reproductive hormones production and sperm quality. These successive key events may result in impaired male fertility (AO). This AOP study summarizes complex data from different biological levels in the literature. It could serve to predict male fertility impairment caused by some NMs using the proposed methods of KE evaluation. Moreover, since the use of the AOP approach is emerging in the nanotoxicology community, proposing some putative AOPs like this one and linking AOPs as networks would help increasing the improvement of mechanistic understanding of pathways interactions involved in various reproductive disorders. Finally, it should be considered that among the factors contributing to the global population decline we are foreseeing, the fertility outcomes related to the decrease in testosterone level and semen quality and quantity, also induced by environmental pollutants, are of pivotal importance. Thus, more efforts should be devoted in the future to better characterize the risk of using new potential endocrine disrupting compounds, as well as to guarantee the better strategies to develop safer and more environmentally sustainable Ag-based materials.

## Supplementary Information

**Additional file 1**. List of the 48 studies selected for the putative AOP construction.

## Data Availability

The datasets used and/or analysed during the current study are available from the corresponding author on reasonable request.

## Abbreviations

Ag NPs: Silver nanoparticles
AO: Adverse outcome
AOP: Adverse outcome pathway
ASINA: Anticipating Safety Issues at the Design Stage of NAno Product Development
BTB: Blood-testis barrier
CAT: Catalase
ESR: Electron spin resonance
FSH: Follicle-stimulating hormone
GSH: Glutathione
HPLC: High performance liquid chromatography
Hsd17b: 17β-Hydroxysteroid dehydorgenase
Hsd3b: 3β-Hydroxysteroid dehydorgenase
KE: Key event
KER: Key Event Relationship
LH: Luteinizing hormone
MDA: Malondialdehyde
MIE: Molecular initiating event
MMP: Mitochondrial membrane potential
MTT: 3-(4,5-Dimethylthiazol-2-yl)-2,5-diphenyltetrazolium bromide
NM: Nanomaterial
NP: Nanoparticle
nm: Nanometer
OECD: The Organisation for Economic Co-operation and Development
pAOP: Putative Adverse Outcome Pathway
PARP: Poly(ADP-ribose) polymerase
PPARα: Peroxisome Proliferator-Activated Receptor alpha
PUFA: Polyunsaturated fatty acids
PVP: Polyvinyl pyrrolidone
ROS: Reactive oxygen species
SbD: Safe by Design
SOD: Super oxide dismutase
StAR: Steroidogenic acute regulatory protein
TBARS: Thiobarbituric acid reactive substances
TM3: Leydig cells
TM4: Sertoli cells
ToxRTool: Toxicological data Reliability Assessment Tool
TSPO: Translocator protein
