# Supplementary material for: Putative adverse outcome pathways for silver nanoparticle toxicity on mammalian male reproductive system: a literature review
Source: Part Fibre Toxicol. 2023 Jan 5;20:1. doi: 10.1186/s12989-022-00511-9 (PMC9814206; doi:10.1186/s12989-022-00511-9)
Supplement: Supplementary file 1 — Additional file 1. List of the 48 studies selected for the putative AOP construction. [file 12989_2022_511_MOESM1_ESM.docx]

**Additional file 1.** List of the 48 studies selected for the putative AOP construction

| **Ag NP**  **p-chem** | **Experimental model and mode of exposure** | **Exposure dose, sampling time** | **Biological/ toxicological**  **Endpoints** | **Actual measurements and methods** | **Result** | **Potential key event(s) (KE nr. from AOP-Wiki) *^a^*** | **Potential adverse outcomes (AOs) associated with the key event (AO nr. from AOP-Wiki*^a^*** | **Ref** |
| --- | --- | --- | --- | --- | --- | --- | --- | --- |
| ***IN VIVO*** | | | | | | | | |
| Size:50 nm  Shape: Spherical  Hydrodynamic size :113.4±12.1 nm  Zeta potential: −12.30 ± 0.4 mV | Adult male SD rats, Oral gavage | 50 mg/kg bw, 3 months | Sperm Evaluation | Sperm Motility, Concentration, and Viability by eosin staining | Increased sperm morphological abnormalities  Decreased sperm concentrations motility  and viability | Decreased sperm quantity or quality in the adult, Decreased fertility (ID505, ID520) | Impaired fertility  (ID330 , ID406) | (1) |
|  |  |  | Oxidative Status | GSH level, CAT activity and lipid peroxidation MDA content in testicular tissue by commercial kits | Decreased CAT activity,  Increased MDA content,  Non-significant change in GSH level | Decreased protection against oxidative stress, Occurance oxidative stress (ID210, ID1112, ID1249, ID1538, ID1869)  Lipid Peroxidation (ID1445 or ID1511) | Oxidative Damage  (ID356) |  |
|  |  |  | Hormonal Assessment | Serum testosterone, LH and FSH by ELISA kit | Decreased testosteron, LH and FSH levels | Reduction, testosterone level (ID1613, ID1689, ID1612)  Reduced, Gonadotropins (ID1986) | Decreased sperm quantity or quality in the adult, Decreased fertility |  |
|  |  |  | DNA damage | DNA strand breaks in testicular tissue by COMET assay | Increased DNA damage | Increased DNA damage (ID1194) | DNA Damage (ID1194) |  |
|  |  |  | Histopathological Examination | Examination of testis and seminiferous tubules by Hematoxylin and Eosin (HE) staining | Histological alterations  Necrotic spermatogonial cells | Testicular atrophy  (ID1506) | Male reproductive tract malformations (ID348)  Reduced, Reproductive Success (ID675) |  |
| Size:20 nm  PVP (0.3%) | Adult male SD rats, Intravenous | 5 mg/kg bw, 15 days (Dosing :3 day intervals) | Sperm Evaluation | Sperm Motility by a microscope and Viability by eosin-nigrosin staining | No significant change | - | - | (2) |
|  |  |  | Mithocondrial functions | Mitochondrial fission protein concentration (DRP1)  Fission-related Mfn1 Mfn2 and OPA1 and Fusion‑related Drp1 gene expressions by RT‑PCR | No significant change  =  No mitochondrial dysfunction | _ | _ |  |
|  |  |  | AgNPs Accumulation | Presence in Testes by ICP | Accumulation in mouse testes | _ | _ |  |
| Size : 19.5 ± 5 nm, Shape : Irregular | Twenty-four male mice (SWR/J), intraperitoneally | 40 mg/kg of bw 35 days of the weekly injection | Sperm Evaluation | Sperm count and motility | Decreased sperm count, total and progressive motility and increasing in immotile sperm | Decreased sperm quantity or quality in the adult, Decreased fertility (ID505, ID520) | Impaired fertility  (ID330 , ID406) |  |
|  |  |  | Hormonal Assessment | Serum testosterone | No alteration in testosteron level | **-** | **-** |  |
|  |  |  | Oxidative Status | TBARS, GSH, CAT, SOD and GST | increased TBARS level , decreased the GSH, GST, SOD and CAT | Decreased protection against oxidative stress, Occurance oxidative stress (ID210, ID1112, ID1249, ID1538, ID1869)  Lipid Peroxidation (ID1445 or ID1511) | Oxidative Damage  (ID356) | (3) |
|  |  |  | Histopathological Examination | Testis and Seminiferous tubules | Changed seminiferous tubules diameters in the testis, apoptotic cells in testes | Testicular atrophy  (ID1506) | Male reproductive tract malformations (ID348)  Reduced, Reproductive Success (ID675) |  |
| Size:32 nm | Male Balb/c mice (n :45, 5–6 weeks old) , Oral gavage | 30 or 125 mg/kg bw , 3months (everyday) | Sperm Evaluation | Sperm motility, and Concentration | Decrease sperm concentrations, and motility (125mg/kg) | Decreased sperm quantity or quality in the adult, Decreased fertility (ID505, ID520) | Impaired fertility  (ID330 , ID406) | (4) |
|  |  |  | Hormonal Assessment | Serum testosterone, LH and FSH by commercial kits | No change in hormon levels | - | - |  |
|  |  |  | Apoptosis | Apoptosis-related gene, oxidative stress, p53 signalling pathway by qRT-PCR | Changes in apoptosis-related gene,  Oxidative stress,  Cell cycle, P53 signalling pathway  gene expression | Apoptosis (ID 1368, 1713, 2075) | Reduce, Sperm count (ID1757)  Testicular atrophy (ID1506)  Reproductive failure (ID1277) |  |
|  |  |  |  | Cell apoptosis in testis by TUNEL | Apoptotic nuclei in spermatogonia and spermatocytes in the testis (125 mg/kg) |  |  |  |
|  |  |  |  | Apoptosis-related proteins Caspase3 and Myc by Immunofluorescence | Up regulation of caspase3 and Myc |  |  |  |
|  |  |  | Histopathological Examination | Histological changes in the testis by HE staining | Histological changes in the testis, including disordered structure, irregular cellular arrangement and increases in intercellular space. Swollen mitochondria in the germ cells ( at 125 mg/kg) | Testicular atrophy  (ID1506) | Male reproductive tract malformations (ID348)  Reduced, Reproductive Success (ID675) |  |
| Size:40 nm  Spherical | Adult male NMRI mice , Oral gavage | 500 mg/kg bw with a time interval of 24 hr for 35 days | Oxidative Status | Total antioxidant capacity and Lipid peroxidation parameters | Decrease in the total antioxidant capacity, Increased MDA concent | Decreased protection against oxidative stress, Occurance oxidative stress (ID210, ID1112, ID1249, ID1538, ID1869)  Lipid Peroxidation (ID1445 or ID1511) | Oxidative Damage  (ID356) | (5) |
|  |  |  | Hormonal Assessment | Serum testosterone level by comercial kits | Decreased testosterone hormone | Reduction, testosterone level (ID1613, ID1689, ID1612) | Decreased sperm quantity or quality in the adult, Decreased fertility |  |
|  |  |  | Histological parametres | Testis, the volume of interstitial tissue and seminiferous tubules | Decreased mean volume of testicular tissue and the volume of seminiferous tubules Decreased sperm density, mean number of spermatocytes, mean number of Sertoli cells | Testicular atrophy  (ID1506) | Male reproductive tract malformations (ID348)  Reduced, Reproductive Success (ID675) |  |
| AgNPs obtained by green synthesis with *Viburnum opulus* L. Size : 17 nm, Shape : Spherical | Ten-week-old pregnant Wistar rats  Intraperitoneally | Two different doses of AgNPs (0.8 and 1.5 mg/kg b.w.) were administered to Wistar female rats on days 3–14 of gestation. Evaluation after 6 weeks of birth. AgNPs D1 group (from mothers treated with 0.8 mg/kg b.w. AgNPs); AgNPs D2 group (from mothers treated with 1.5 mg/kg b.w. AgNPs). | Oxidative Status | Blood and testicular tissue MDA, GSH, and GSH/GSSG level | Increased MDA content,  GSH/GSSG ratio AgNPs D2 group | Decreased protection against oxidative stress, Occurance oxidative stress (ID210, ID1112, ID1249, ID1538, ID1869)  Lipid Peroxidation (ID1445 or ID1511) | Oxidative Damage  (ID356) | (6) |
|  |  |  | Apoptosis , DNA lesions | p53, BCL2, BAX and γH2Ax proteins in the testes | Decreased p53 BAX protein , no change in Bcl-2 , γH2Ax analysis displayed significantly higher values in the AgNPs D2 group | Mitochondrial dependent Apoptosis | Reduce, Sperm count (ID1757)  Testicular atrophy (ID1506)  Reproductive failure (ID1277) |  |
|  |  |  | *Inflammation and transcription factor NFkB/NFkB expressions in the testis* | TNFα and IL1α expressions and transcription factor NFkB | Decreased TNFα and IL1α  Increased NFkB expression significantly in the AgNPs D1  Even if the nanoparticles persisted locally, the inflammation induced by them was minimal, probably due to their green synthesis | **-** | - |  |
|  |  |  | Determination of tissue Ag | Ag presence in testes AAS | NPs exceed the blood-testicular barriers and accumulated in the testis, where they triggered important morphofunctional changes that persisted long after the exposure was ended. | Penetration to the blood–testis barrier | Testicular atrophy  (ID1506) |  |
|  |  |  | Histological parametres | Testes, the seminiferous tubules and interstitial tissue evaluation by TEM | Important ultrastructural changes of Sertoli cells, numerous vacuoles and cytoplasmic changes suggestive of the cellʹs evolution towards necrosis | Testicular atrophy  (ID1506) | Male reproductive tract malformations (ID348)  Reduced, Reproductive Success (ID675) |  |
| Size:45 nm  PVP (<1%)  Zeta potential:−20 mV | New Zealand White male rabbits, Intravenous | 5 mM AgNP solution (0.6 mg/kg bw) 2 months (weekly) | Sperm Evaluation | Sperm Volume, Concentration, and Viability | Decreased sperm motility, concentration and volume | Decreased sperm quantity or quality in the adult, Decreased fertility | Impaired fertility |  |
|  |  |  | Oxidative Status | MDA content, NO concentration, CAT and GPX Activity in sperm and blood samples by commercial kits | Increased NO, and MDA content  Decreased CAT activity, and GSH level | Decreased protection against oxidative stress, Occurance oxidative stress (ID210, ID1112, ID1249, ID1538, ID1869)  Lipid Peroxidation (ID1445 or ID1511) | Oxidative Damage  (ID356) | (7) |
|  |  |  | Cytokine assessment | IL-6, IL-8, and TNF-α in seminal plasma by the Bio-Plex Cytokine assay | Increased cytokines | Increased Pro-inflammatory mediators (ID1493) | Inflammation (ID 149, 1087, 1633) |  |
| Size:100 nm  Spherical  SSA : 7.5329m^2^/g  Zeta potential:− 18.9 mV | Male Rats, Sub dermal | 10 and 50 mg/kg bw, 7 and 28 days | Sperm Evaluation | Sperm motility, velocity by HE staining | Decreased sperm motility and velocity | Decreased sperm quantity or quality in the adult, Decreased fertility (ID505, ID520) | Impaired fertility  (ID330 , ID406) | (8) |
|  |  |  | Oxidative Status | MDA, GSH, CAT | Increased Lipid peroxidization Decreased SOD, CAT, GSH and total thiols | Decreased protection against oxidative stress, Occurance oxidative stress (ID210, ID1112, ID1249, ID1538, ID1869)  Lipid Peroxidation (ID1445 or ID1511) | Oxidative Damage  (ID356) |  |
|  |  |  | Hormonal Assessment | Testosterone, LH and FSH | Decreased testosterone, LH and FSH levels (dose dependent) | Reduction, testosterone level (ID1613, ID1689, ID1612)  Reduced, Gonadotropins (ID1986) | Decreased sperm quantity or quality in the adult, Decreased fertility |  |
|  |  |  | Histological parametres | In testicular samples | Degenerative alterations in the cellular architecture of testes and epididymis | Testicular atrophy  (ID1506) | Male reproductive tract malformations (ID348)  Reduced, Reproductive Success (ID675) |  |
| Size:10, 20, 40, 60 and 100 nm  Hydrodynamic size : 113.4±12.1 nm | Male mice (BALB/C), intraperitoneal route | 1 mg/kg bw 35 days | Histological parametres | Cellular achitecture of testes and epididymis | Degenerative spermatogenic cells, mild intertubular oedema, spermatocytes desquamation and spermatid giant cell | Testicular atrophy  (ID1506) | Male reproductive tract malformations (ID348)  Reduced, Reproductive Success (ID675) | (9) |
| Size:<100 nm, Shape: Spherical, Zeta Potential : -28 mV, Sigma-Aldrich 7440-22-4  Hydrodynamic size : 215 nm (PDI of 0.41)  Zeta potential:− 20 mV | Male Wistar rats, intratesticular injection | AgNPs solution (0.46 µg-Ag/ml) in each testicle, 7, 14, 28, and 56 days | Sperm Evaluation | Sperm Motility, morphology | Decrease sperm motility decrease in the percentage of normal spermatozoa, increase in abnormal morphology (D7) | Decreased sperm quantity or quality in the adult, Decreased fertility (ID505, ID520) | Impaired fertility  (ID330 , ID406) | (10) |
|  |  |  | Histological parametres | Tubule and tubular lumen diameter and area, together with seminiferous epithelium height | All the tubular parameters significantly reduced in the AgNPs-D7 group | Testicular atrophy  (ID1506) | Male reproductive tract malformations (ID348)  Reduced, Reproductive Success (ID675) |  |
| Size:100 nm | Wistar rats, intraperitoneal | 100 mg/kg bw, 8 weeks | Oxidative Status | GSH, TBARS, GST, SOD in testicular tissue | Decreased SOD, GST level, increased TBARS | Decreased protection against oxidative stress, Occurance oxidative stress (ID210, ID1112, ID1249, ID1538, ID1869)  Lipid Peroxidation (ID1445 or ID1511) | Oxidative Damage  (ID356) | (11) |
|  |  |  | Hormonal Assessment | Serum testosterone, LH and FSH | Decreased testosteron, LH and FSH levels | Reduction, testosterone level (ID1613, ID1689, ID1612)  Reduced, Gonadotropins (ID1986) | Decreased sperm quantity or quality in the adult, Decreased fertility |  |
|  |  |  | DNA damage | DNA fragmentation in testicular tissue | Increase in testicular DNA fragmentation | Increased DNA damage (ID1194) | DNA Damage (ID1194) |  |
|  |  |  | Histopathological Examination | Testis morphology, seminiferous tubule area, circumference, and diameter | Disturbance in the spermatogenic cells arrangements, atrophied seminiferous tubules with degenerative Sertoli cell, marked decrease in the number of sperms, depletion in Leydig cells | Testicular atrophy  (ID1506) | Male reproductive tract malformations (ID348)  Reduced, Reproductive Success (ID675) |  |
| Size:30 nm Shape:polygonal,round, and triangular | Female Wistar rats, intraperitoneally | 0.156 mg/mL 0.625 mg/mL  The administration for all the groups was performed once a day on two continuous days in early and late gestations (E5, E6, E13, and E14) | Histopathological Examination | Testis morphology, seminiferous tubule area, circumference, and diameter | Significant difference seminiferous tubules in the Leydig cells, mild inflammation and hyperemia in the testis | Testicular atrophy  (ID1506) | Male reproductive tract malformations (ID348)  Reduced, Reproductive Success (ID675) | (12) |
| AgNPs were prepared using the bakers yeast  (*S. cerevisiae*)  Size : 20.0 ±1.3 nm  Shape : Spherical | SDrats, oral route | 50 mg/kg/day daily for 8 weeks | Sperm Evaluation | Motility, viability, concentration, sperm abnormalities | Reduction in sperm motility, epididymal sperm count live/dead ratio, and elevation in sperm abnormalities | Decreased sperm quantity or quality in the adult, Decreased fertility (ID505, ID520) | Impaired fertility  (ID330 , ID406) | (13) |
|  |  |  | Oxidative Status | MDA,  GPx, SOD, and CAT by commerical kits | increased MDA level , decreased GPx, SOD, and CAT activity | Decreased protection against oxidative stress, Occurance oxidative stress (ID210, ID1112, ID1249, ID1538, ID1869)  Lipid Peroxidation (ID1445 or ID1511) | Oxidative Damage  (ID356) |  |
|  |  |  | Hormonal Assessment  hypothalamic–pituitary–gonadal axis | Serum levels of FSH, LH and testosterone by ELISA methods and expression of hypothalamic GnRH1, testicular AR by RT-PCR | Reduced expression of GnRH1, AR, and serum levels of FSH, LH and testosterone and increased hypothalamic AR suggesting downregulation of the HPG axis. | Reduction, testosterone level (ID1613, ID1689, ID1612)  Reduced, Gonadotropins (ID1986) | Decreased sperm quantity or quality in the adult, Decreased fertility (ID505, ID520) |  |
|  |  |  | Determination of tissue Ag and Blood–testis barrier (BTB) permeability | Ag presence in testes AAS, Tight junctions proteins (occludin, claudin-11, and tight junction protein 1 (Tjp1)) and transforming growth factor (TGF)-β | NP accumulation in testes, Reduced expression level of tight junction proteins and TGF-β | Increased blood–testis barrier (BTB) permeability | Testicular atrophy  (ID1506) |  |
|  |  |  | Histological parametres | Epididymal and testicular | Atrophy of seminiferous tubules, thinning of the tubule wall, and disorganization and vacuolization of germinal epithelium, and loss of spermatogenic cells in testi | Decreased protection against oxidative stress, Occurance oxidative stress (ID210, ID1112, ID1249, ID1538, ID1869)  Lipid Peroxidation (ID1445 or ID1511) | Oxidative Damage  (ID356) |  |
| Size:60-80 nm Shape: Spherical Hydrodynamic size : 250nm | Wistar rats, intraperitoneal | 30, 125 and 300 mg/kg bw  4 weeks | Sperm Evaluation | Sperm Morphology, count, Motility, viability | Decreased sperm vitality   and sperm count, normal sperm morphology (at 300 mg/kg) | Decreased sperm quantity or quality in the adult, Decreased fertility | Impaired fertility | (14) |
|  |  |  | Histological parametres | Testis morphology, seminiferous tubule area, circumference, and diameter | Reduction in the number of spermatogonia, Sertoli and Leydig cells (125 and 300 mg/kg)  Reduction in the diameter, circumference and mean area of seminiferous tubules (125 mg/kg) | Testicular atrophy  (ID1506) | Male reproductive tract malformations (ID348)  Reduced, Reproductive Success (ID675) |  |
| Size: 8.93-33.4 nm Shape: Spherical Hydrodynamic size : 197.4 ± 2.7 and 422.4 ± 6.3 Zeta potential −33.6 and −37.5 mV | SD rats, Oral gavage | 5.36 mg/kg, 13.4 mg/kg twice weekly for six months | Sperm Evaluation | Sperm Morphology, count, Motility, viability, Sperm DNA Damage by Sperm chromatin integrity Acridine orange (AO) | Decreased sperm vitality   and sperm count, normal sperm morphology, Decrease in DNA chromatin integrity | Decreased sperm quantity or quality in the adult, Decreased fertility (ID505, ID520) Increased DNA damage (ID1194) | DNA Damage (ID1194), Impaired fertility  (ID330 , ID406) | (15) |
|  |  |  | Oxidative Status | SOD and MDA in testicular tissue by commercial kits | Decreased SOD, increased MDA | Decreased protection against oxidative stress, Occurance oxidative stress (ID210, ID1112, ID1249, ID1538, ID1869)  Lipid Peroxidation (ID1445 or ID1511) | Oxidative Damage  (ID356) |  |
|  |  |  | Hormonal Assessment | Serum testosterone, LH and FSH by ELISA kits | Decreased testosterone hormone, increased the level of LH at the 4^th^, 5^th^ and 6^th^ month, no FSH level change | Reduction, testosterone level (ID1613, ID1689, ID1612) | Decreased sperm quantity or quality in the adult, Decreased fertility |  |
|  |  |  | Histopathology | Histopathology of testes by TEM | The spermatozoa decreases and have missed head shape  The interstitial cells mostly appeared inactive, have a large vesicular nucleus and few cell organelles in the cytoplasm. | Testicular atrophy  (ID1506) | Male reproductive tract malformations (ID348)  Reduced, Reproductive Success (ID675) |  |
| Size:60 nm, Sigma-Aldrich 730815 | Newly weaned male Wistar rats, Oral gavage | 1.875, 3.750, 7.500, or 15 μg of AgNP/kg bw PND23 to PND60 | Oxidative Status (testicular tissue) | GPX, GSR, and CAT activity by commercial kits | Increased GPX CAT activity, Decreased GSR activity | Decreased protection against oxidative stress, Occurance oxidative stress (ID210, ID1112, ID1249, ID1538, ID1869) | Oxidative Damage  (ID356) | (16,17) |
|  |  |  |  | Sod1, Cat, Gsr expression by RT-qPCR | Decreased, Gsr mRNA content, Sod1 transcript was reduced, Increased Cat transcript |  |  |  |
|  |  |  | Sperm evaluation | Sperm function: acrosome integrity, plasma membrane integrity, mitochondrial activity | Reduced acrosome integrity (15 μg) unchanged the plasma membrane integrity and the sperm morphology Decreased the mitochondrial activity ( 7.5 and 15 μg) | Decreased sperm quantity or quality in the adult, Decreased fertility (ID505, ID520) | Impaired fertility  (ID330 , ID406) |  |
| Size: 21 - 41 nm (PVP coated), Shape: Spherical Hydrodynamic size : 90 nm in FBS Zeta potential : 135 mV in FBS | BALB/c mice, intraperitoneally | 0.25, 0.5 and 1 mg Ag NPs/kg bw  9 day | Sperm Evaluation | Sperm morphology, concentation, motility by eosin-nigrosin staining | Increased sperm morphological abnormalities dose dependent Decrease sperm viablity and motility | Decreased sperm quantity or quality in the adult, Decreased fertility (ID505, ID520) | Impaired fertility  (ID330 , ID406) | (18) |
|  |  |  | Oxidative Status (testicular tissue) | Total antioxidant capacity (TAC) level, MDA level | Increased MDA, decreased TAC level | Occurance oxidative stress (ID210, ID1112, ID1249, ID1538, ID1869)  Lipid Peroxidation (ID1445 or ID1511) | Oxidative Damage  (ID356) |  |
|  |  |  | Histopathology | Histopathology of testes | Few necrotic spermatogonial cells at 0.25 mg/kg Ag NPs; moderate testicular degeneration and necrosis of spermatogonial cells 0.50 mg/kg Ag NPs; and severe testicular degeneration and complete necrosis of spermatogonial cells 1 mg/kg Ag NPs. | Testicular atrophy  (ID1506) | Male reproductive tract malformations (ID348)  Reduced, Reproductive Success (ID675) |  |
|  |  |  |  |  |  |  |  |  |
| Size: <100 nm Sigma-Aldrich 7440-22-4 | Wistar albino rats, intraperitoneal | 100 mg/kg/day and 1000 mg/kg/day for 7 days, 7 days/week, for 28 days. | Sperm Evaluation | Sperm count, motility, viability and morphology by eosine nigrosin staining | Decrease in the sperm count, motility, viability increase in sperm abnormality (dose and time dependant) | Decreased sperm quantity or quality in the adult, Decreased fertility (ID505, ID520) | Impaired fertility  (ID330 , ID406) | (19) |
|  |  |  | Hormonal assessment | Serum testosterone by commercial kits | Decrease in serum testosterone levels | Reduction, testosterone level (ID1613, ID1689, ID1612) | Decreased sperm quantity or quality in the adult, Decreased fertility |  |
|  |  |  | Spermatogenesis releated gene expressions | Dazl Tnp2 GDNF gene expression by RT-PCR | Down regulation of Dazl, Tnp2 and GDNF | Impaired spermatogenesis (ID1758) | Impaired fertility  (ID406) |  |
|  |  |  | Histological parametres | H&E stained sections of the testes | Necrosis and degradation of spermatogenic cells, and Sertoli cells  Increase in the mean relative testicular weight  Atrophy in seminiferous tubules | Testicular atrophy  (ID1506) | Male reproductive tract malformations (ID348)  Reduced, Reproductive Success (ID675) |  |
| Size: 10, 20, 40, 60 and 100 nm | Male mice (BALB/C) intraperitoneally | 1 and 1000 mg/kg/day 35 days. | Histological parametres | Histological parametres of testes | Slight decrease on the relative ratios of total testis weight to body weight and testis index | - | - | (20) |
| Size: 56.67 ± 9.77 nm, Shape:Cubic, Zeta potential: 9.35mV | Male Swiss Webster mice, intraperitoneally | 0, 20, 41, and 82mg/kg  24 hours | Sperm Evaluation | Sperm Motility, Concentration | Induced sperm abnormality, decreased sperm concentrations and sperm motility | Decreased sperm quantity or quality in the adult, Decreased fertility (ID505, ID520) | Impaired fertility  (ID330 , ID406) | (21) |
|  |  |  | Oxidative Status | MDA and CAT in testis and sperm | Increased MDA content, Decrease CAT activity | Decreased protection against oxidative stress, Occurance oxidative stress (ID210, ID1112, ID1249, ID1538, ID1869)  Lipid Peroxidation (ID1445 or ID1511) | Oxidative Damage  (ID356) |  |
|  |  |  | DNA Damage | DNA damage in testes by COMET assay | Increased DNA damage | Increased DNA damage (ID1194) | DNA Damage (ID1194) |  |
| Size:20 ± 5 nm and 200 ± 50 nm, Shape: Spherical, Zeta potential −33.6 ± 5.59 mV and −37.5 mV ± 5.18 mV | Adult male Wistar rats, Intravenous | 20 nm AgNPs in two different doses (5 mg/kg body weight (b.w.) (Ag I group) and 10 mg/kg b.w. (Ag II group)) and 200 nm AgNPs at a dose of 5 mg/kg bw (Ag III group) | Sperm Evaluation | Sperm Motility, and concentration by eosin staining | Decrease sperm concentrations and sperm motility | Decreased sperm quantity or quality in the adult, Decreased fertility (ID505, ID520) | Impaired fertility  (ID330 , ID406) | (22,23) |
|  |  |  | Hormonal Assessment | LH, plasma total T and E2 hormone, DHT by radioimmunoassay and commercial kits | Decrease androgen concentrations time dependent Increased plasma LH and E2 concentration Decreased Plasma T and DHT concentrations | Reduction, testosterone level (ID1613, ID1689, ID1612) | Decreased sperm quantity or quality in the adult, Decreased fertility |  |
|  |  |  | Intratesticular steroid metabolism enzyme protein level | Aromatase (Aro) and 5α-reductase type 1 (Srd5a1) measurements by commercial ELISA kits | A progressive decrease in the intratesticular levels of androgens and proteins | Decrease, Steroidogenic acute regulatory protein (STAR) (ID 436) | Reduction, Cholesterol transport in mitochondria (ID447)  Reduction, testosterone level (ID1613, ID1689, ID1612) |  |
|  |  |  | Steroidogenesis and steroid metabolism gene expression | Expression of Star,Cyp11a1, Hsd3b1 and Hsd17b3 by qPCR | Genes related to sper-matogenesis were down-regulated |  |  |  |
| 20 nm | ICR Mice, intravenous | 0.5mg/kg and 1 mg/kg of AgNPs | Expression of cytokines | TNF-α, IFN-γ, IL-6, IL-1β and MCP-1 genes expressions | Increased TNF-α, IFN-γ, IL-6, IL-1β and MCP-1 expression level | Increased Pro-inflammatory mediators (ID1493) | Inflammation (ID 149, 1087, 1633) | (24) |
|  |  |  | Apoptosis | Apoptosis on testes by TUNEL assay | Total atrophy of all seminiferous tubules in the testis, apoptosis in  spermatogonia including spermatocytes and spermatids | Testicular atrophy  (ID1506) | Male reproductive tract malformations (ID348)  Reduced, Reproductive Success (ID675) |  |
|  |  |  | Steroidogenesis and steroid metabolism gene expression | 3 β-hsd, and 17β-hsd, Cyp17a1 and Cyp19a1 expression | Decreased the expression levels of 3β-hsd, and 17β-hsd, Cyp17a1 and Cyp19a1 | Inhibition the synthesis of male steroid hormones | Reduction, testosterone level (ID1613, ID1689, ID1612) |  |
| PVP-coated AgNPs Size: 20-30 nm Shape:Spherical | SD rats, oral gavage | 0, 50, 100 and 200 mg/kg bw 90 days, daily dose | Sperm Evaluation | Sperm Count, Motility, Morphology, Viability by ofeosin–nigrosin staining | No change motiliy, count and viability , increase in abnormal sperm formations, being significant at 100 mg/kg. | **-** | **-** | (25) |
|  |  |  | Histological parametres | Epididymal and testicular, leydig cells by HE staining | No significant changes in testes morphology  No significant alterations of epididymal ducts, vacuolization on epithelium,or cell alterations | **-** | **-** |  |
| Size: 60 nm | Weaned male Wistar rats  Oral gavage | 15 or 50 μg/kg/day PNDs 23–58 exposure, PND 102 observation | Sperm evaluation | Sperm function: acrosome integrity plasma membrane integrity, mitochondrial activity by Cytochrome c oxidase | Reduced acrosome and plasma membrane integrity, increased sperm abnormalities. Reduced mitochondrial activity | Decreased sperm quantity or quality in the adult, Decreased fertility (ID505, ID520) | Impaired fertility  (ID330 , ID406) | (26) |
|  |  |  | Hormonal Assessment | FSH, LH, testosterone and estradiol | No differences vs. control | - | - |  |
| Size:50-60 nm, Shape: Spherical, Hydrodynamic size :113.4±12.1 nm, Zeta potential: −12.30 ± 0.4 mV | Albino mice intraperitoneal | 0.5, 1, 5 mg/kg bw 35 days | Sperm evaluation | Sperm Motility, and count, chromatin condensation by Eosin–nigrosin and aniline-blue staining | Decrease sperm count, sperm chromatin condensations, motility | Decreased sperm quantity or quality in the adult, Decreased fertility (ID505, ID520) | Impaired fertility  (ID330 , ID406) | (27) |
|  |  |  | Oxidative Status | TAOC by ferric reduction antioxidant power (FRAP) assay and MDA by thethiobarbituric acid (TBA) reaction assay in testicular tissue | Decreased TAOC level, increased MDA content, | Decreased protection against oxidative stress, Occurance oxidative stress (ID210, ID1112, ID1249, ID1538, ID1869)  Lipid Peroxidation (ID1445 or ID1511) | Oxidative Damage  (ID356) |  |
|  |  |  | Hormonal Assessment | Serum testosterone, LH and FSH by Radioimmunoassay kit | Reduction, testosterone level LH and FSH level | Reduction, testosterone level (ID1613, ID1689, ID1612) | Decreased sperm quantity or quality in the adult, Decreased fertility |  |
|  |  |  | Histological parametres | Histological parametres of testes | Reduction in Leydig cells distribution. Increased the percentage of hypertrophied Leydig cells, Decreased testis index, seminiferous tubules atrophy | Testicular atrophy  (ID1506) | Male reproductive tract malformations (ID348)  Reduced, Reproductive Success (ID675) |  |
| Size: 20 and 200 nm, Shape: Spherical, Hydrodynamic size :158.5 ± 2.2, 326.4 ± 5.5 | Male 8-oxoguanine DNA glycosylase knockout mice (Ogg1 / KO^-1-^), intravenous | 5 mg/kg BW, 1 to 7 days | Sperm evaluation | Sperm chromatin structure assay | No significant change in sperm DNA fragmentation | - | - | (28) |
|  |  |  | DNA damage | Comet assay in testicular tissue | Increased DNA damage (on 7th day) | DNA damage (ID 1468) | Apoptosis (ID 1365) |  |
|  |  |  |  | 21 gene expression in DNA damage response, DNA repair, antioxidant enzymes by qPCR | Up-regulation of genes in DNA damage response/repair pathway, in antioxidant response |  |  |  |
| *Sythesized method : from Bacillus funiculus*, Size 15 nm, Shape:Spherical | Eight-day-old (PND8) male mice abdominal sub­cutaneous injection | 13 days (from PND8 to PND21) at 1 and 5 mg/kg per dose. Observation : PND28, PND42, PND63, and PND100 | Sperm evaluation | Morphology and concentration  Sperm quality associated *DDx3Y* and *E1F1AY* genes expressions by RT-PCR | Abnormal sperm morphology at PND42, PND60, and PND100  Reduced sperm concentration at 5 mg/kg at PND100  The *DDx3Y* and *E1F1AY* genes were up-regulated at PND28 and PND42 but not at PND60 | Decreased sperm quantity or quality in the adult, Decreased fertility (ID505, ID520) | Impaired fertility  (ID330 , ID406) | (29) |
| Citrate-coated AgNPs, Size: 10.4 ± 1.9 nm | CD1 mice, intravenous | 1 mg/kg bw over 12 days | Sperm Evaluation | Sperm Motility, and Concentration | No change in motility and concentration | - | - | (30) |
|  |  |  | Hormonal Assessment | Serum testosterone, LH and FSH | No change in LH and FSH, Increased, testosterone level (only 15th day) | - | - |  |
|  |  |  | Steroidogenesis and steroid metabolism gene expression | Steroidogenic acute regulatory protein (StAR), Translocator protein (TSPO), Cyp11a1 , Cyp17a1, Hsd17b3,and Cyp19a1, Growth hormone and insulin-like growth factor 1 (IGF1) expression by RT-PCR | No change in steroid biosynthesis increase in Cyp11a1 levels and transcripts for Hsd3b1 in the testes, No significant change in IGF1 | - | - |  |
|  |  |  | Histological parametres | Testes parameters | Increased Lumen volume and tubule diameter on day 15 and 60; % apoptotic germ cells on days 15,60, and 120  Decreased seminiferous epithelium volume density on days 15 and 60. Decreased Seminiferous epithelium volume density | Testicular atrophy  (ID1506) | Male reproductive tract malformations (ID348)  Reduced, Reproductive Success (ID675) |  |
| Size:5-20 nm Shape:Spherical | SD rats, Oral gavage | 20µg/kg/day, 90 days | Histopathological Examination | Testes parameters | Atrophy of seminiferous tubules, Necrotic spermatogenic cells; ultra structural changes inspermatogonia Leydig and Sertoli cells | Testicular atrophy  (ID1506) | Male reproductive tract malformations (ID348)  Reduced, Reproductive Success (ID675) | (31) |
| Size:70 nm | Male wistar rats, Oral gavage | 25, 50, 100, 200 mg/kg bw, 45 days | Sperm Evaluation | Sperm Morphology, and Motility | Abnormal morphology, reduced percentage of normal spermatozoa Decreased motility, | Decreased sperm quantity or quality in the adult, Decreased fertility (ID505, ID520) | Impaired fertility  (ID330 , ID406) | (32) |
|  |  |  | Hormonal Assessment | Serum testosterone, LH and FSH | Reduction, testosterone level  Increase, LH  Non-significant reduction in FSH | Reduction, testosterone level (ID1613, ID1689, ID1612) | Decreased sperm quantity or quality in the adult, Decreased fertility |  |
|  |  |  | Histopathological Examination | Testes parameters | Reduced Leydig cell numbers  Seminiferous tubular atrophy, Necrotic spermatogenic cells, Testes atrophy | Testicular atrophy  (ID1506) | Male reproductive tract malformations (ID348)  Reduced, Reproductive Success (ID675) |  |
| Size:21 – 41 nm, Shape:Spherical | Male mice intraperitoneal | 100, 500 and 1000 mg/kg for 28 days | Sperm Evaluation | Sperm Morphology, Motility, Count, Viability, DNA integrity | Abnormal morphology, Decrease sperm motility, viability, integrity and quality (500 and 1000 mg/kg) | Decreased sperm quantity or quality in the adult, Decreased fertility (ID505, ID520) | Impaired fertility  (ID330 , ID406) | (33) |
|  |  |  | Oxidative Status | MDA and SOD in testes | Decreased SOD activity, Increased MDA (500 and 1000 mg/kg) | Decreased protection against oxidative stress, Occurance oxidative stress (ID210, ID1112, ID1249, ID1538, ID1869)  Lipid Peroxidation (ID1445 or ID1511) | Oxidative Damage  (ID356) |  |
|  |  |  | Hormonal Assessment | Serum testosterone | Reduction, testosterone level (500 and 1000 mg/kg) | Reduction, testosterone level (ID1613, ID1689, ID1612) | Decreased sperm quantity or quality in the adult, Decreased fertility |  |
|  |  |  | Histopathological Examination |  | Disruption in the testicular architecture and loss in spermatogenic stages, testicular atrophy and loss in height of spermatogenic epithelium | Testicular atrophy  (ID1506) | Male reproductive tract malformations (ID348)  Reduced, Reproductive Success (ID675) |  |
| Size:45 nm  Zeta potential: -20 mV | New Zealand White male rabbits, intravenous | 0.6 mg/kg bw single dose. Rabbit testicular tissue and semen were obtained from rabbits killed at days 21, 42, 84, 105, and 126 after NP treatment | Sperm evaluation | Sperm morphology and Acrosome reaction (AR), Curvilinear velocity (VCL), Ag NP presence in sperm | Decreased Sperm motility; VCL, % of normal sperm; sperm oxygen consumption.  Increased % of AR sperm; ultrastructural sperm damage,  AgNPs in cytoplasmic residues, sections of nucleus, acrosome of semen samples (On days 7–126, except day 42) | Decreased sperm quantity or quality in the adult, Decreased fertility (ID505, ID520) | Impaired fertility  (ID330 , ID406) | (34) |
|  |  |  | Histopathological Examination |  | No histopathological changes were observed in testes. Normal morphology of Sertoli and Leydig cells, and spermatids. | **-** | **-** |  |
| Size:10-20 nm  Shape: Spherical  Zeta potential: -15 mV | Adult male SD rats, intratracheal instillation | 50 µg/rat single dose, observation at day 7, or 28 | Oxidative stress in testes | Oxidative stress-related genes (*Gpx1, SOD, FMO2* and *GAPDH)*, metal toxicity (*Mt1*), apoptosis/cell cycle (*casp3*, *p53*), and protein-folding processes (*Hsp70*) | Increased expression of genes involved in oxidative stress (GPX1, SOD, FMO2, and GAPDH) in testes on day 7, but not day 28 | **-** | **-** | (35) |
| Size:70 nm | Male Wistar rats, Oral gavage | 25, 50, 100, or200 mg/kg/day 48 days (every 12 h) | Sperm evaluation | Morphology, count and acrosome reaction, | Abnormal morphology, Decreased sperm count, impaired acrosome reaction | Decreased sperm quantity or quality in the adult, Decreased fertility (ID505, ID520) | Impaired fertility  (ID330 , ID406) | (36) |
|  |  |  | Histological assessment | Testes parameters | Damaged testis tubules | Testicular atrophy  (ID1506) | Male reproductive tract malformations (ID348)  Reduced, Reproductive Success (ID675) |  |
| Size: 60 nm | Thirty newly weaned male Wistar rats  Oral gavage | PND53 until PND90  15, or 50 μg*/*kg bw | Sperm Evaluation | Sperm reserves, sperm transit time, sperm production | Decreased sperm reserves in the epididymis and diminished  Sperm transit time at PND53.  Reduction in the total and daily sperm production at PND90 | Decreased sperm quantity or quality in the adult, Decreased fertility (ID505, ID520) | Impaired fertility  (ID330 , ID406) | (37) |
|  |  |  | Hormonal Assessment | Serum testosterone, estradiol | No alteration testosterone estradiol serum concentrations | **-** | **-** |  |
|  |  |  | Histopathological Examination | Testis and Seminiferous tubules | No significant change | **-** | **-** |  |
| AgNPs in the 0.9% citrate solution, Size: 10 nm and 25 nm | Male SD rats, Oral gavage | 100 or 500 mg/kg/day over 28 days  Observation : At the end of exposure or 1, 2, or 4 months after ceasing exposure | Determination of tissue Ag | Ag presence in testes AAS | Ag NPs accumulation in testes  No histopathological changes in testes. | - | - | (38) |
| AgNPs (AS: 17.7 nm) and PVP-AgNPs(AS: 12.2 nm, 75% PVP coating | Male SD rats,Oral Gavage | 90 mg/kg/day over 28 days AgNPs and PVP-AgNPs  Oservation : day 29, 36, 84 | Determination of tissue Ag | Ag presence in testes | High Ag levels in testes | Increased blood–testis barrier (BTB) permeability | Testicular atrophy  (ID1506) | (39) |
| Size : 56 nm  Shape : Spherical | Fisher 344 rats, Oral gavage | 13 week 0, 125, or500 mg/kg/day | Determination of tissue Ag | Ag presence in testes AAS | Dose-dependent increase in testicular Ag levels | Penetration to the blood–testis barrier | Testicular atrophy  (ID1506) | (40) |
| AgNPs (Sigma–Aldrich 484059)  Size : 22, 42, 71, or 323 nm | Male ICR mice, Oral gavage | 1 mg/kg/day over 14 days | Determination of tissue Ag | Ag presence in testes AAS | NP accumulation in testes | Increased blood–testis barrier (BTB) permeability | Testicular atrophy  (ID1506) | (41) |
| Size : 20, 80 and 110 nm, Shape: Spherical | Wistar rats, intravenous | 1 mL nanosilver dispersion once daily for 5 consecutive days. | Determination of tissue Ag | Ag presence in testes AAS | NP accumulation in testes | Increased blood–testis barrier (BTB) permeability | Testicular atrophy  (ID1506) | (42) |
| ***IN VITRO*** | | | | | | | | |
| *Sythesized method : from Bacillus funiculus*, Size 15 nm, Shape:Spherical | TM3 and TM4 cell lines | 0-50 µg/ml for 24 h exposure | Cytotoxicity | MTT and LDH assay | Decreased cell viability, and increased LDH leakage | Increased cytotoxicity (ID887, ID2659) | Increase, Tissue Degeneration, Necrosis & Atrophy (ID868) | (29) |
| AgNPs coated with 0.2% PVP Size : 30 nm | Human sperm | 0, 50 μg/ml, 100 μg/ml, 200 μg/ml or 400 μg/ml for 15 min, 30 min, or 60 min exposure | Sperm Evaluation | Motility, viability, concentration, sperm abnormalities | AgNP exposure exerted significant time- and dose-dependent spermatotoxic effects | Decreased sperm quantity or quality in the adult, Decreased fertility (ID505, ID520) | Impaired fertility, (ID330 , ID406) | (43) |
|  |  |  | Oxidative status and DNA Damage | ROS production and DNA fragmentation | AgNPs increased ROS production and the ratio of fragmented to normal DNA in human sperm, indicating that AgNP-induced toxicity in human spermatozoa involved ROS stress and DNA damage. | Oxidative stress (ID1690), Increase DNA damage (ID1897) | Increase, Apoptosis (ID1365) |  |
| 20 nm | Sertoli (TM4) cells | 0, 3.0625, 6.125, 12.5, 25 and 50 mg/mL for 24 h exposure | Cytotoxicity | Cell viability by CCK-8 and LDH Assay | Decreased viability, increased LDH release | Increased cytotoxicity (ID887, ID2659) | Increase, Tissue Degeneration, Necrosis & Atrophy (ID868) | (24) |
|  |  |  | Oxidative status | ROS Production by H2DCF-DA assay | AgNPs induce the production of ROS | Oxidative stress (ID1690) | Increase, Apoptosis (ID1365) |  |
|  |  |  | Apoptosis | TUNEL Assay | Nuclear and morphological changes are characteristics of apoptosis | General Apoptosis (ID1513) | Testicular atrophy (ID1506) |  |
| 10, 40 and 100 nm | Sertoli cells (15P-1) | 0.5, to 15.0 μg/ml or 24 h exposure | Cytotoxicity | Cell membrane integrity and permeability (NRU assay), and the mitochondrial metabolic activity (MTT assay) | All kinds of AgNPs showed strong cytotoxic activity | increase cytotoxicity (ID887, ID2659) | Increase, Tissue Degeneration, Necrosis & Atrophy (ID868) | (44) |
|  |  |  | Oxidative Status | Lipid peroxidation assessments by TBARS, Total antioxidative status | Increase in TBARS, increase in antioxidative potential | Decreased protection against oxidative stress, Occurance oxidative stress (ID210, ID1112, ID1249, ID1538, ID1869) Lipid Peroxidation (ID1445 or ID1511) | General Apoptosis (ID1513) , Oxidative Damage  (ID356) |  |
| 10 nm and 20 nm | Leydig (TM3) and Sertoli (TM4) cells | 10 µg/ml for 24h exposure | Cytotoxicity | Cell viability by MTS assay, CCK-8 Assay and LDH Assay | Inhibited the viability, proliferation and Cell membrane integrity of cells. AgNPs-reduced cell viability was due to ROS generation and that accumulated ROS caused apoptosis of cells | increase cytotoxicity (ID887, ID2659) | Increase, Tissue Degeneration, Necrosis & Atrophy (ID868) | (45) |
|  |  |  | Oxidative status | ROS Production by H2DCF-DA assay | The upregulation of intracellular ROS production | Oxidative stress (ID1690) | Cell injury/death (ID55), General Apoptosis (ID1513) , Oxidative Damage  (ID356) |  |
|  |  |  | Apoptosis | Annexin V/propidium iodide staining | increase in cellular apoptosis | General Apoptosis (ID1513) | Testicular atrophy (ID1506) |  |
|  |  |  | Steroidogenesis and steroid metabolism gene expression | levels of StAR, 3β-Hsd, 17β-Hsd, luteinizing hormone receptor (LhR), and Cyp17a1 | AgNPs inhibits StAR, 3β-HSD, and 17β-HSD, Cyp17a1 transcription | Decrease, Steroidogenic acute regulatory protein (STAR) (ID 436),Reduction, testosterone level (ID1613, ID1689, ID1612) | Reduction, testosterone level (ID1613, ID1689, ID1612), Impaired fertility (ID330 , ID406) |  |
| 54.8 nm, −53.33 ± 7.86 mV. | chicken sertoli cells | 25, 75 and 125 ppm nanosilver for 48 h. | Cytotoxicity | Cell viability by trypan blue exclusion | The percentage of live cells progressively decreased with increasing nanosilver concentration | increased cytotoxicity (ID887, ID2659) | Increase, Tissue Degeneration, Necrosis & Atrophy (ID868) | (46) |
|  |  |  | Oxidative status | SOD and NOS genes expression by RT-PCR, Lipid peroxidation by TBARS Assay | Reduced expression of SOD genes and increased expression of NOS genes, Lipid peroxidation was significantly increased | Decreased protection against oxidative stress, Occurance oxidative stress (ID210, ID1112, ID1249, ID1538, ID1869) Lipid Peroxidation (ID1445 or ID1511) | General Apoptosis (ID1513) , Oxidative Damage  (ID356) |  |
| 20 and 200 nm | Ntera2 (NT2, human testicular embryonic carcinoma cell line), and primary testicular cells from C57BL6 mice of wild type (WT) and 8-oxoguanine DNA glycosylase  knock-out (KO, mOgg1−/−) genotype | 10, 50, 100 µg/ml, | Cytotoxicity | Determination of cell metabolic activity by the MTT assay | At 24 h and at maximum (µg/ml) AgNP concentration, the metabolic activity of all three cell types was reduced to approximately 50% of controls. | increased cytotoxicity (ID887, ID2659) | Increase, Tissue Degeneration, Necrosis & Atrophy (ID868) | (47) |
|  |  |  | DNA Damage | DNA-strand breaks by COMET assay | Ag200 caused the highest level of damage, with about 25% DNA intensity in tail at the 100 µg ml−1 concentration, whereas Ag20 caused approximately 15% strand breakage in NT2 cells. In contrast, the NPs caused little to no DNA-strand breaks in testicular cells derived from either WT or KO (Ogg1−/−) | Increase DNA damage (ID1897) | Increase, Apoptosis (ID1365) |  |
| 15 nm | C18–4 cell line | 5, 10, 25, 50, and 100 µg/ml exposure | Cytotoxicity | Mitochondrial function by MTT assay, membrane integrity by LDH assay | Reduced mitochondrial function and cell viability. A slight increase in LDH leakage, indicating that these particles interfere with cell metabolism rather than disrupting the plasma membrane. Thus, they might promote cell apoptosis rather than necrosis | increase cytotoxicity (ID887, ID2659) | Increase, Apoptosis (ID1365) | (48) |

1. Shehata AM, Salem FMS, El-Saied EM, Abd El-Rahman SS, Mahmoud MY, Noshy PA. Zinc nanoparticles ameliorate the reproductive toxicity induced by silver nanoparticles in male rats. Int J Nanomedicine. 2021;16:2555–68.

2. Arslan NP, Keles ON, Gonul-Baltaci N. Effect of Titanium Dioxide and Silver Nanoparticles on Mitochondrial Dynamics in Mouse Testis Tissue. Biol Trace Elem Res. 2021;(0123456789).

3. Abu-Taweel GM, Albetran HM, Al-Mutary MG, Ahmad M, Low IM. Alleviation of silver nanoparticle-induced sexual behavior and testicular parameters dysfunction in male mice by yttrium oxide nanoparticles. Toxicol Reports [Internet]. 2021;8:1121–30. Available from: https://doi.org/10.1016/j.toxrep.2021.05.014

4. Wang E, Huang Y, Du Q, Sun Y. Alterations in reproductive parameters and gene expression in Balb/c mice testes after exposure to silver nanoparticles. Andrologia. 2021;53(1):1–9.

5. Shariatzadeh SMA, Miri SA, Cheraghi E. The protective effect of Kombucha against silver nanoparticles-induced toxicity on testicular tissue in NMRI mice. Andrologia. 2021;53(3):1–10.

6. Bidian C, Filip GA, David L, Florea A, Moldovan B, Robu DP, et al. The impact of silver nanoparticles phytosynthesized with Viburnum opulus L. extract on the ultrastrastructure and cell death in the testis of offspring rats. Food Chem Toxicol. 2021;150(February).

7. Collodel G, Simona M, Moretti E, Cerretani D, Lucia M, Anna Ida F, et al. Oxidative and/or Inflammatory Thrust Induced by Silver Nanoparticles in Rabbits: Effect of Vitamin E or NSAID Administration on Semen Parameters. Mediators Inflamm. 2020;2020.

8. Olugbodi JO, David O, Oketa EN, Lawal B, Okoli BJ, Mtunzi F. Silver nanoparticles stimulates spermatogenesis impairments and hematological alterations in testis and epididymis of Male rats. Molecules [Internet]. 2020 Feb 27 [cited 2021 Jun 29];25(5). Available from: https://pubmed.ncbi.nlm.nih.gov/32120976/

9. Al-Doaiss AA, Jarrar Q, Alshehri M, Jarrar B. In vivo study of silver nanomaterials’ toxicity with respect to size. Toxicol Ind Health. 2020;36(8):540–57.

10. de Brito JLM, Lima VN de, Ansa DO, Moya SE, Morais PC, Azevedo RB de, et al. Acute reproductive toxicology after intratesticular injection of silver nanoparticles (AgNPs) in Wistar rats. Nanotoxicology [Internet]. 2020;14(7):893–907. Available from: https://doi.org/10.1080/17435390.2020.1774812

11. Altwaijry N, El-Masry TA, Alotaibi B, Tousson E, Saleh A. Therapeutic effects of rocket seeds (Eruca sativa L.) against testicular toxicity and oxidative stress caused by silver nanoparticles injection in rats. Environ Toxicol. 2020;35(9):952–60.

12. Pourali P, Nouri M, Ameri F, Heidari T, Kheirkhahan N, Arabzadeh S, et al. Histopathological study of the maternal exposure to the biologically produced silver nanoparticles on different organs of the offspring. Naunyn Schmiedebergs Arch Pharmacol. 2020;393(5):867–78.

13. Arisha AH, Ahmed MM, Kamel MA, Attia YA, Hussein MMA. Morin ameliorates the testicular apoptosis, oxidative stress, and impact on blood–testis barrier induced by photo-extracellularly synthesized silver nanoparticles. Environ Sci Pollut Res. 2019;26(28):28749–62.

14. Fathi N, Hoseinipanah SM, Alizadeh Z, Assari MJ, Moghimbeigi A, Mortazavi M, et al. The effect of silver nanoparticles on the reproductive system of adult male rats: A morphological, histological and DNA integrity study. Adv Clin Exp Med. 2019;28(3):299–305.

15. Elsharkawy EE, Abd El-Nasser M, Kamaly HF. Silver nanoparticles testicular toxicity in rat. Environ Toxicol Pharmacol [Internet]. 2019 Aug 1 [cited 2021 Jun 29];70. Available from: https://pubmed.ncbi.nlm.nih.gov/31255771/

16. Lopes IMD, De Oliveira IM, Bargi-Souza P, Cavallin MD, Kolc CSMH, Khalil NM, et al. Effects of Silver Nanoparticle Exposure to the Testicular Antioxidant System during the Prepubertal Rat Stage. Chem Res Toxicol. 2019;32(6):986–94.

17. Cavallin MD, Wilk R, Oliveira IM, Cardoso NCS, Khalil NM, Oliveira CA, et al. The hypothalamic-pituitary-testicular axis and the testicular function are modulated after silver nanoparticle exposure. Toxicol Res (Camb) [Internet]. 2018;7(1):102–16. Available from: http://dx.doi.org/10.1039/c7tx00236j

18. Moradi-Sardareh H, Basir HRG, Hassan ZM, Davoudi M, Amidi F, Paknejad M. Toxicity of silver nanoparticles on different tissues of Balb/C mice. Life Sci. 2018 Oct 15;211:81–90.

19. Ahmed SM, Abdelrahman SA, Shalaby SM. Evaluating the effect of silver nanoparticles on testes of adult albino rats (histological, immunohistochemical and biochemical study). J Mol Histol. 2017;48(1):9–27.

20. Almansour M, Jarrar Q, Battah A, Jarrar B. Histomorphometric Alterations Induced in the Testicular Tissues by Variable Sizes of Silver Nanoparticles - PubMed [Internet]. J Reprod Med . 2017 [cited 2021 Jun 30]. Available from: https://pubmed.ncbi.nlm.nih.gov/30027728/

21. Mohamed HRH. Studies on the Genotoxicity Behavior of Silver Nanoparticles in the Presence of Heavy Metal Cadmium Chloride in Mice. J Nanomater. 2016;2016.

22. Dziendzikowska K, Krawczyńska A, Oczkowski M, Królikowski T, Brzóska K, Lankoff A, et al. Progressive effects of silver nanoparticles on hormonal regulation of reproduction in male rats. Toxicol Appl Pharmacol. 2016;313:35–46.

23. Gromadzka-Ostrowska J, Dziendzikowska K, Lankoff A, Dobrzyńska M, Instanes C, Brunborg G, et al. Silver nanoparticles effects on epididymal sperm in rats. Toxicol Lett. 2012;214(3):251–8.

24. Han JW, Jeong JK, Gurunathan S, Choi YJ, Das J, Kwon DN, et al. Male- and female-derived somatic and germ cell-specific toxicity of silver nanoparticles in mouse. Nanotoxicology. 2016;10(3):361–73.

25. Lafuente D, Garcia T, Blanco J, Sánchez DJ, Sirvent JJ, Domingo JL, et al. Effects of oral exposure to silver nanoparticles on the sperm of rats. Reprod Toxicol [Internet]. 2016;60:133–9. Available from: http://dx.doi.org/10.1016/j.reprotox.2016.02.007

26. Mathias FT, Romano RM, Kizys MML, Kasamatsu T, Giannocco G, Chiamolera MI, et al. Daily exposure to silver nanoparticles during prepubertal development decreases adult sperm and reproductive parameters. Nanotoxicology [Internet]. 2015 Feb 1 [cited 2021 Jun 29];9(1):64–70. Available from: https://pubmed.ncbi.nlm.nih.gov/24533579/

27. Rezazadeh-Reyhani Z, Razi M, Malekinejad H, Sadrkhanlou R. Cytotoxic effect of nanosilver particles on testicular tissue: Evidence for biochemical stress and Hsp70-2 protein expression. Environ Toxicol Pharmacol [Internet]. 2015;40(2):626–38. Available from: http://dx.doi.org/10.1016/j.etap.2015.08.024

28. Asare N, Duale N, Slagsvold HH, Lindeman B, Olsen AK, Gromadzka-Ostrowska J, et al. Genotoxicity and gene expression modulation of silver and titanium dioxide nanoparticles in mice. Nanotoxicology [Internet]. 2016 [cited 2021 Jun 29];10(3):312–21. Available from: https://www.tandfonline.com/action/journalInformation?journalCode=inan20http://informahealthcare.com/

29. Zhang X-F, Gurunathan S, Kim JH. Effects of silver nanoparticles on neonatal testis development in mice. Int J Nanomedicine. 2015;10:6243–56.

30. Garcia TX, Costa GMJ, França LR, Hofmann MC. Sub-acute intravenous administration of silver nanoparticles in male mice alters Leydig cell function and testosterone levels. Reprod Toxicol. 2014;45:59–70.

31. Thakur M, Gupta H, Singh D, Mohanty IR, Maheswari U, Vanage G, et al. Histopathological and ultra structural effects of nanoparticles on rat testis following 90 days (Chronic study) of repeated oral administration. J Nanobiotechnology. 2014;12(1):1–13.

32. Baki ME, Miresmaili SM, Pourentezari M, Amraii E, Yousefi V, Spenani HR, et al. Effects of silver nano-particles on sperm parameters, number of Leydig cells and sex hormones in rats. Iran J Reprod Med. 2014;12(2):139–44.

33. Attia AA. Evaluation of the testicular alterations induced by silver nanoparticles in male mice: biochemical, histological and ultrastructural studies. Res J Pharm Biol Chem Sci. 2014;5(4):1558–89.

34. Castellini C, Ruggeri S, Mattioli S, Bernardini G, Macchioni L, Moretti E, et al. Systems Biology in Reproductive Medicine Long-term effects of silver nanoparticles on reproductive activity of rabbit buck Long-term effects of silver nanoparticles on reproductive activity of rabbit buck. Syst Biol Reprod Med [Internet]. 2014 [cited 2021 Jun 29];60(3):143–50. Available from: https://www.tandfonline.com/action/journalInformation?journalCode=iaan20http://informahealthcare.com/aan

35. Coccini T. Gene Expression Changes in Rat Liver and Testes after Lung Instillation of a Low Dose of Silver Nanoparticles. J Nanomed Nanotechnol. 2014;05(05).

36. Miresmaeili SM, Halvaei I, Fesahat F, Fallah A, Nikonahad N, Taherinejad M. Evaluating the role of silver nanoparticles on acrosomal reaction and spermatogenic cells in rat. Iran J Reprod Med. 2013;11(5):423–30.

37. Sleiman HK, Romano RM, Oliveira CA De, Romano MA. Effects of prepubertal exposure to silver nanoparticles on reproductive parameters in adult male wistar rats. J Toxicol Environ Heal - Part A Curr Issues. 2013;76(17):1023–32.

38. Lee J, Kim Y, Song K, Ryu H, Sung J, Park J, et al. Biopersistence of silver nanoparticles in tissues from Sprague-Dawley rats. Part Fibre Toxicol [Internet]. 2013 Aug 1 [cited 2021 Jul 16];10(1). Available from: https://pubmed.ncbi.nlm.nih.gov/24059869/

39. van der Zande M, Vandebriel RJ, Doren E Van, Kramer E, Rivera ZH, Serrano-Rojero CS, et al. Distribution, Elimination, and Toxicity of Silver Nanoparticles and Silver Ions in Rats after 28-Day Oral Exposure. ACS Nano [Internet]. 2012 Aug 28 [cited 2021 Jul 16];6(8):7427–42. Available from: https://pubs.acs.org/doi/full/10.1021/nn302649p

40. Soon Kim Y, Yong Song M, Duck Park J, Seuk Song K, Ryol Ryu H, Hyun Chung Y, et al. Subchronic oral toxicity of silver nanoparticles. 2010 [cited 2021 Jul 16]; Available from: http://www.particleandfibretoxicology.com/content/7/1/20

41. Park EJ, Bae E, Yi J, Kim Y, Choi K, Lee SH, et al. Repeated-dose toxicity and inflammatory responses in mice by oral administration of silver nanoparticles. Environ Toxicol Pharmacol. 2010 Sep 1;30(2):162–8.

42. Lankveld DPK, Oomen AG, Krystek P, Neigh A, Troost - de Jong A, Noorlander CW, et al. The kinetics of the tissue distribution of silver nanoparticles of different sizes. Biomaterials. 2010 Nov 1;31(32):8350–61.

43. Wang E, Huang Y, Du Q, Sun Y. Silver nanoparticle induced toxicity to human sperm by increasing ROS(reactive oxygen species) production and DNA damage. Environ Toxicol Pharmacol. 2017 Jun 1;52:193–9.

44. Zapór L. Effects of silver nanoparticles of different sizes on cytotoxicity and oxygen metabolism disorders in both reproductive and respiratory system cells. Arch Environ Prot. 2016;42(4):32–47.

45. Zhang X-F, Choi Y-J, Han JW, Kim E, Park JH, Gurunathan S, et al. Differential nanoreprotoxicity of silver nanoparticles in male somatic cells and spermatogonial stem cells. Int J Nanomedicine [Internet]. 2015 Feb 16 [cited 2021 Jul 19];10:1335–57. Available from: https://pubmed.ncbi.nlm.nih.gov/25733828/

46. Hassanpour H, Mirshokraei P, Khalili Sadrabad E, Esmailian Dehkordi A, Layeghi S, Afzali A, et al. In vitro effect of nanosilver on gene expression of superoxide dismutases and nitric oxide synthases in chicken sertoli cells. Animal [Internet]. 2014;9(2):295–300. Available from: http://dx.doi.org/10.1017/S1751731114002262

47. Asare N, Instanes C, Sandberg W, Refsnes M, Schwarze P, Kruszewski M, et al. Cytotoxic and genotoxic effects of silver nanoparticles in testicular cells. Toxicology [Internet]. 2012 Jan 27 [cited 2021 Aug 10];291(1–3):65–72. Available from: https://pubmed.ncbi.nlm.nih.gov/22085606/

48. Braydich-Stolle L, Hussain S, Schlager J, Hofmann M-C. In vitro cytotoxicity of nanoparticles in mammalian germline stem cells. Toxicol Sci [Internet]. 2005 Dec [cited 2021 Jul 19];88(2):412–9. Available from: https://pubmed.ncbi.nlm.nih.gov/16014736/
